# Palladium-Catalysed Coupling Reactions En Route to Molecular Machines: Sterically Hindered Indenyl and Ferrocenyl Anthracenes and Triptycenes, and Biindenyls

**DOI:** 10.3390/molecules25081950

**Published:** 2020-04-22

**Authors:** Michael J. McGlinchey, Kirill Nikitin

**Affiliations:** School of Chemistry, University College Dublin, Belfield, D04 V1W8 Dublin 4, Ireland

**Keywords:** restricted rotations, M(CO)_3_ tripods, molecular brakes and gears, X-ray, V-T NMR

## Abstract

Pd-catalysed Stille and Suzuki cross-couplings were used to prepare 9-(3-indenyl)-, 6, and 9-(2-indenyl)-anthracene, 7; addition of benzyne led to the 9-Indenyl-triptycenes, 8 and 9. In 6, [4 + 2] addition also occurred to the indenyl substituent. Reaction of 6 through 9 with Cr(CO)_6_ or Re_2_(CO)_10_ gave their M(CO)_3_ derivatives, where the Cr or Re was complexed to a six- or five-membered ring, respectively. In the 9-(2-indenyl)triptycene complexes, slowed rotation of the paddlewheel on the NMR time-scale was apparent in the η^5^-Re(CO)_3_ case and, when the η^6^-Cr(CO)_3_ was deprotonated, the resulting haptotropic shift of the metal tripod onto the five-membered ring also blocked paddlewheel rotation, thus functioning as an organometallic molecular brake. Suzuki coupling of ferrocenylboronic acid to mono- or dibromoanthracene yielded the ferrocenyl anthracenes en route to the corresponding triptycenes in which stepwise hindered rotations of the ferrocenyl groups behaved like molecular dials. CuCl_2_-mediated coupling of methyl- and phenyl-indenes yielded their *rac* and *meso* 2,2′-biindenyls; surprisingly, however, the apparently sterically crowded *rac* 2,2′-Bis(9-triptycyl)biindenyl functioned as a freely rotating set of molecular gears. The predicted high rotation barrier in 9-phenylanthracene was experimentally validated via the Pd-catalysed syntheses of di(3-fluorophenyl)anthracene and 9-(1-naphthyl)-10-phenylanthracene.

## 1. Introduction

**Figure 1:**
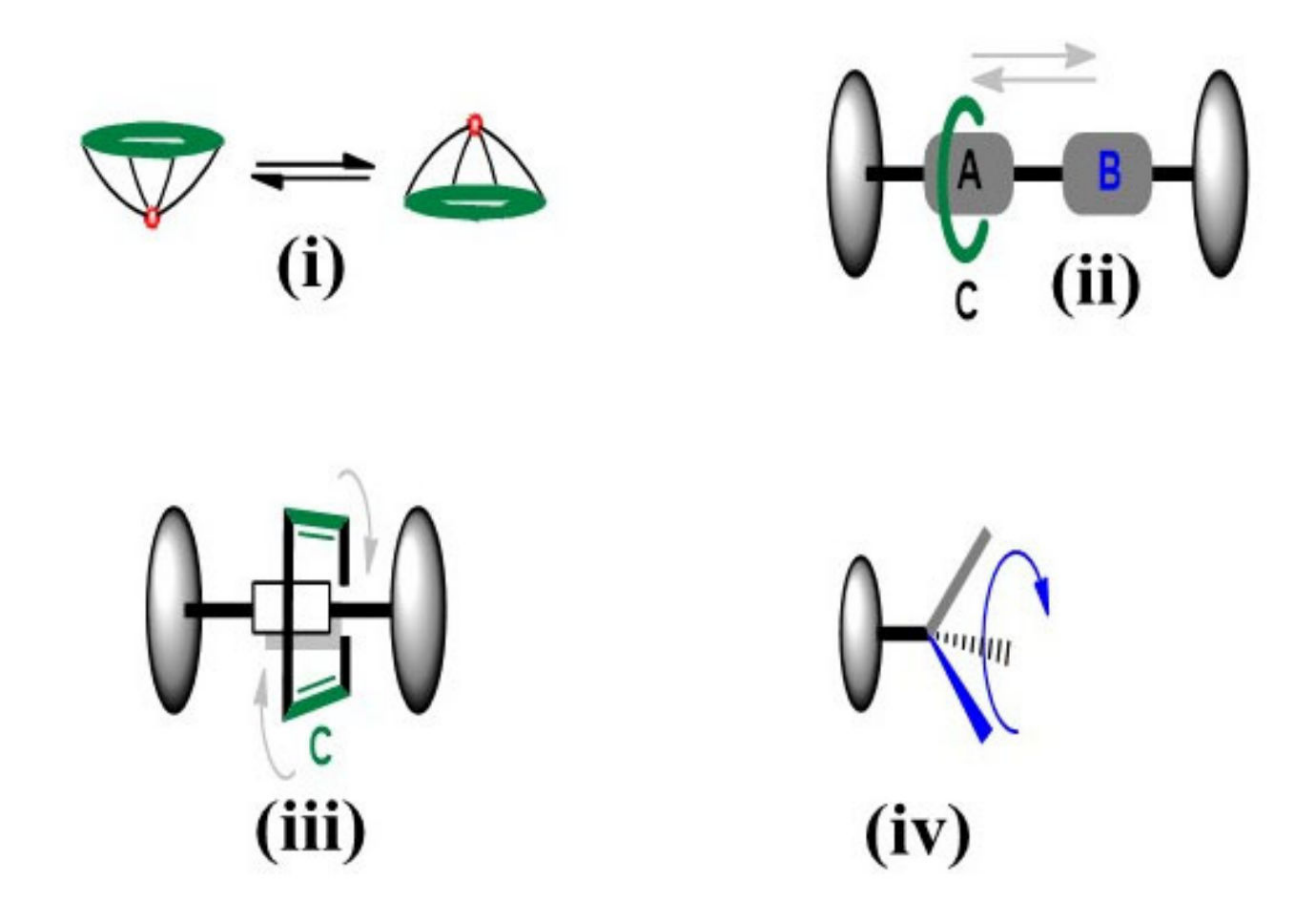
Examples of fluxional molecular systems: (**i**) bowl-to-bowl inversion, (**ii**) shuttling, (**iii**) pirouetting, and (**iv**) rotating.

In recent decades, the goal of creating artificial molecule-scale mechanical systems (e.g., molecular machines, shuttles, gears, rotors, turnstiles) has attracted enormous attention within the synthetic community [1,2]. Herein, we focus on one specifically selected approach-transition metal catalysed coupling [3], towards the synthesis of the “skeletal backbones” of molecular mechanical systems, their basic construction elements. Incorporation of these rigid fragments allows us to investigate the functional behaviour of a wide selection of individual and mechanically interlocked systems, such as those illustrated schematically in Figure 1. Even a brief perusal of the vast literature on the syntheses of molecular machines leads one to conclude that transition metal-catalysed (especially palladium-catalysed) coupling reactions play a prominent role in this area of modern synthetic chemistry, as exemplified below.

**Scheme 1:**
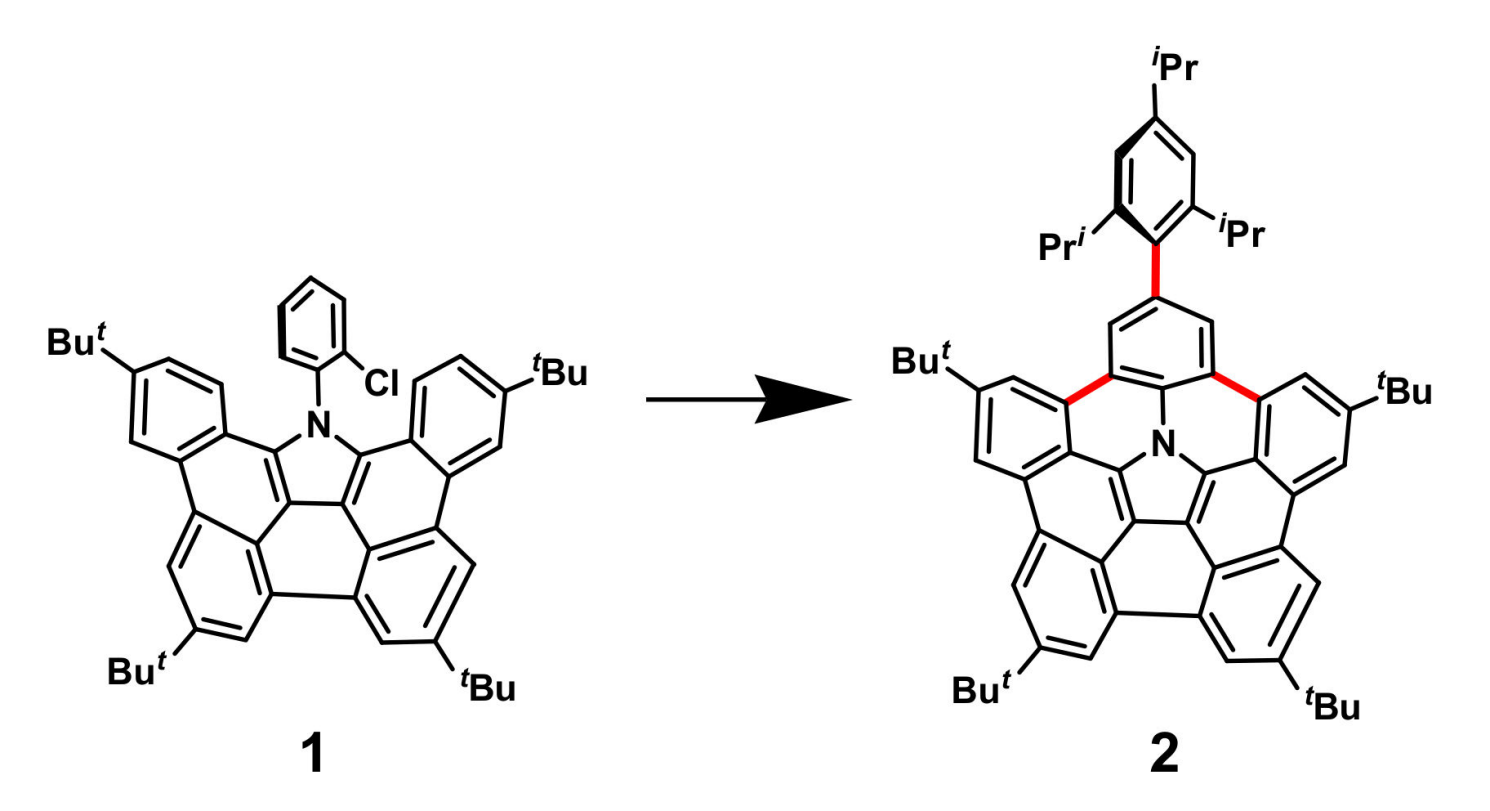
Efficient synthesis of the buckybowl, **2,** via Pd-catalysed C-C bond formation.

Following the pioneering work on corannulene by Siegel et al. [4], the study of an umbrella-type inversion process in the nitrogen-embedded buckybowl, 2, from its precursor, 1, was made possible via three sequential Pd-catalysed coupling reactions (shown in red, Scheme 1). The bowl-to-bowl inversion barrier was reported to be 98 kJ mol^−1^ [5].

**Figure 2:**
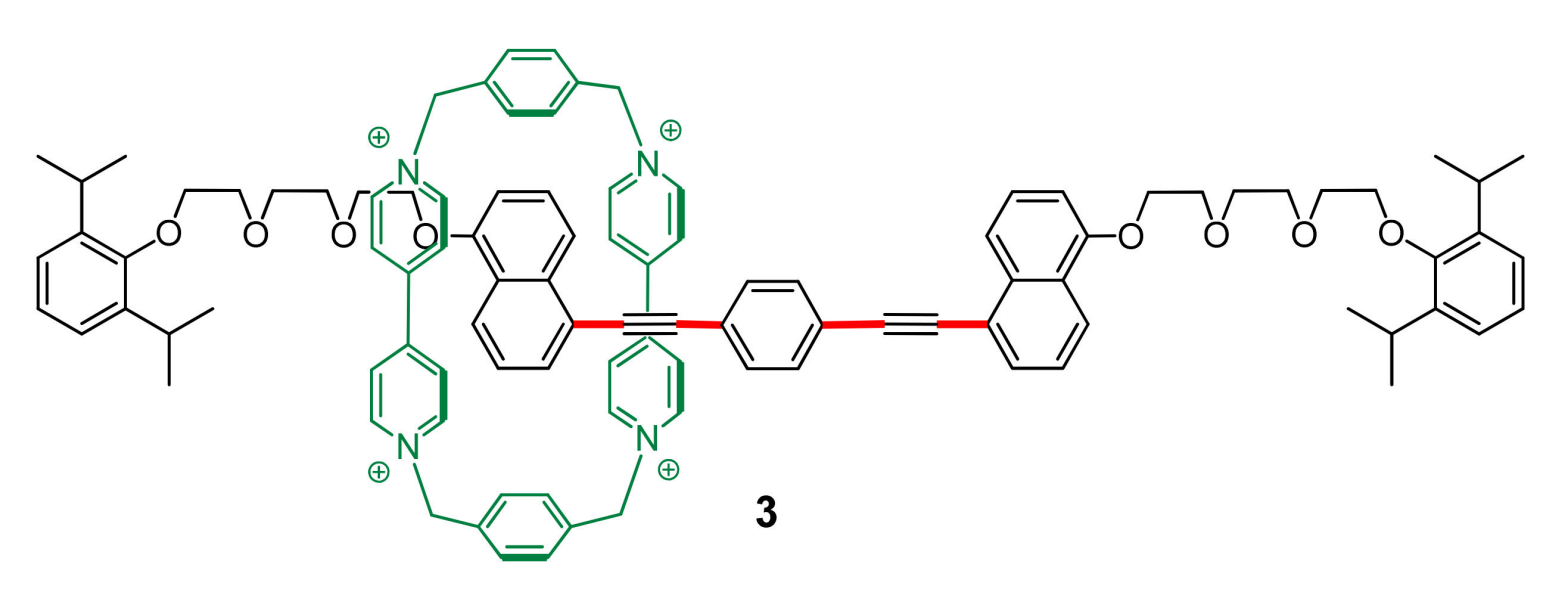
Fast degenerate shuttling of a cyclobis(paraquat-*p*-phenylene) ring along a rigid dialkyne framework.

Palladium-catalysed aryl-acetylene coupling reactions have been used by Stoddart’s group to construct long rigid molecular spacers of the [2]rotaxane shuttle, 3, (Figure 2) whereby low shuttling energy barriers were reported unless a “speed bump” moiety was attached to the naphthalene ring [6].

**Figure 3:**
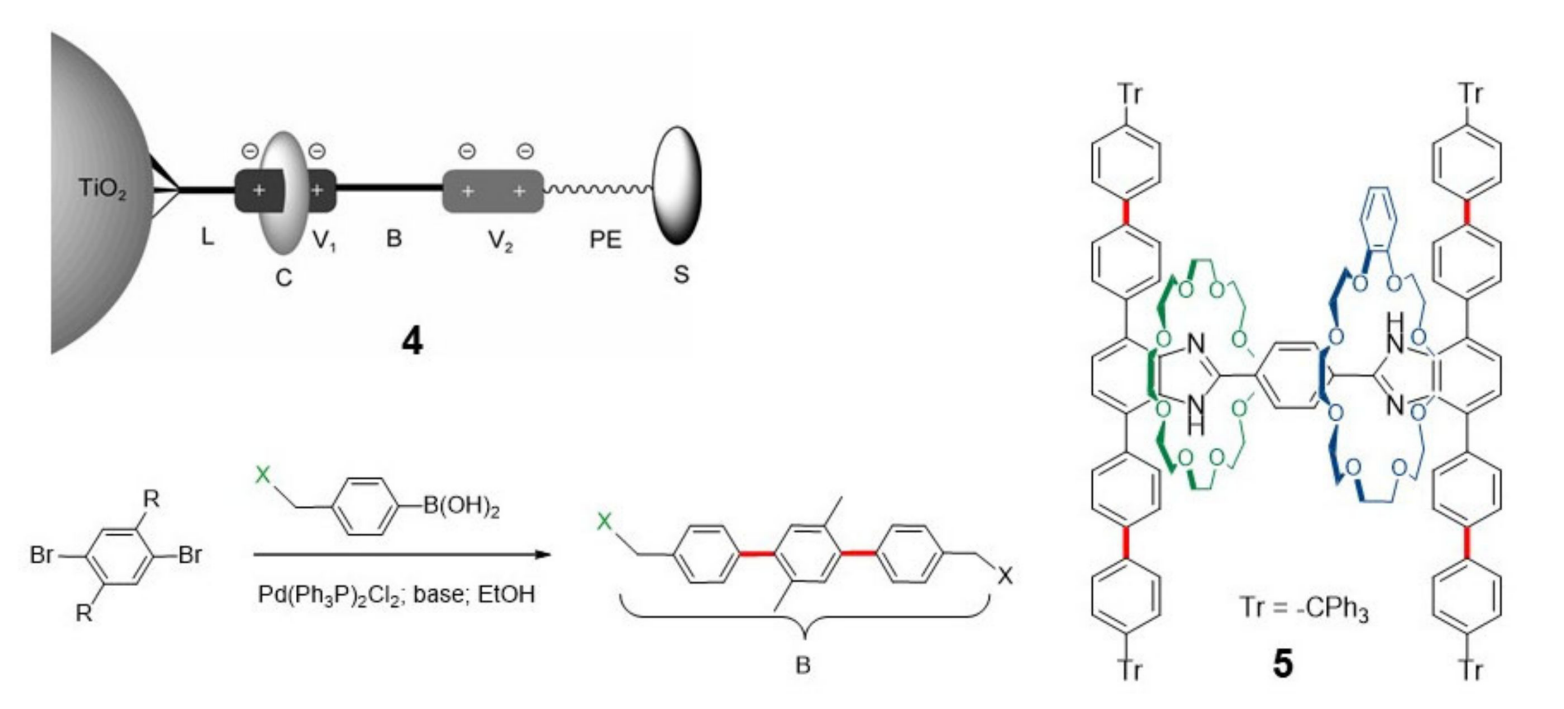
Palladium-catalysed formation of C–C bonds in the tripodal rigid non-degenerate shuttle, **4** [7,8], and the rigid ring-in-ring shuttle, **5** [9].

In a similar vein, we preceded these results with our non-degenerate tripodal two-station rotaxane shuttle, 4 (Figure 3), which was designed to be adsorbed in an oriented way on a titanium dioxide surface [7]. Unexpectedly, the synthesis of the rigid spacer, B, via Suzuki-type cross-coupling proved problematic, as no terphenyl product could be isolated for R = H, X = OH; however, when R = Me, X = OH, the terphenyl linker was isolable in 75% yield, and was fully characterised by X-ray crystallography [8]. Similarly, catalysed coupling reactions were recently used by Loeb and co-workers in the synthesis of oligo-*p*-phenylene components of their solid-state shuttle 5 [9].

**Figure 4:**
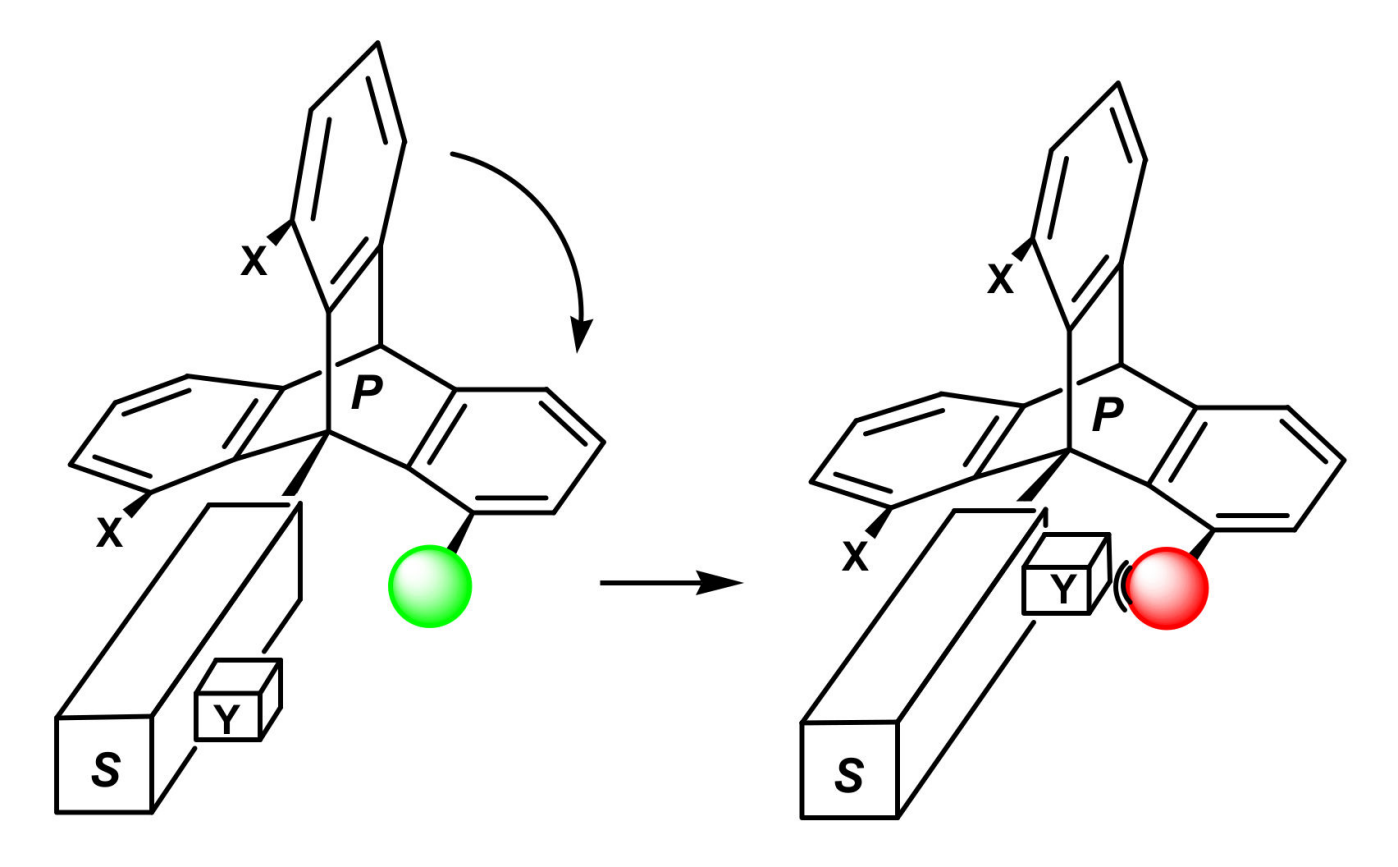
Schematic of a molecular gearing system in which free rotation of the paddlewheel P is controlled by sliding the “latch” Y of the shuttle S.

In the present report, we collect together some of our own work and focus chiefly on molecular machines and rotors, whereby a defined pair of molecular fragments is connected by a rotatable single C-C bond (Figure 1, part iv) to attain restricted intramolecular rotation. Our goal was to prepare molecular gearing systems, incorporating a paddlewheel-shaped triptycene fragment attached to a molecular shuttle. The dynamic behaviour of the shuttle could, in principle, allow free rotation or function as a molecular brake, as in Figure 4.

The planned approach was to prepare 9-(3-indenyl)anthracene, 6, and 9-(2-indenyl)anthracene, 7. The synthesis of such molecules requires the attachment of the bicyclic unit to anthracene, with subsequent Diels-Alder addition of benzyne to form the corresponding indenyl triptycenes, 8 and 9, respectively. One could then envisage incorporation of a bulky organometallic fragment that could undergo a reversible η^6^ ⇿ η^5^ haptotropic shift across the indenyl framework, whereby the pentahapto-complexed system would hinder paddlewheel rotation. An appropriate cross-coupling procedure appeared to offer the most propitious route to 6 and 7.

## 2. Indenyl Anthracenes and Triptycenes

### 2.1. Synthetic Aspects

**Scheme 2:**
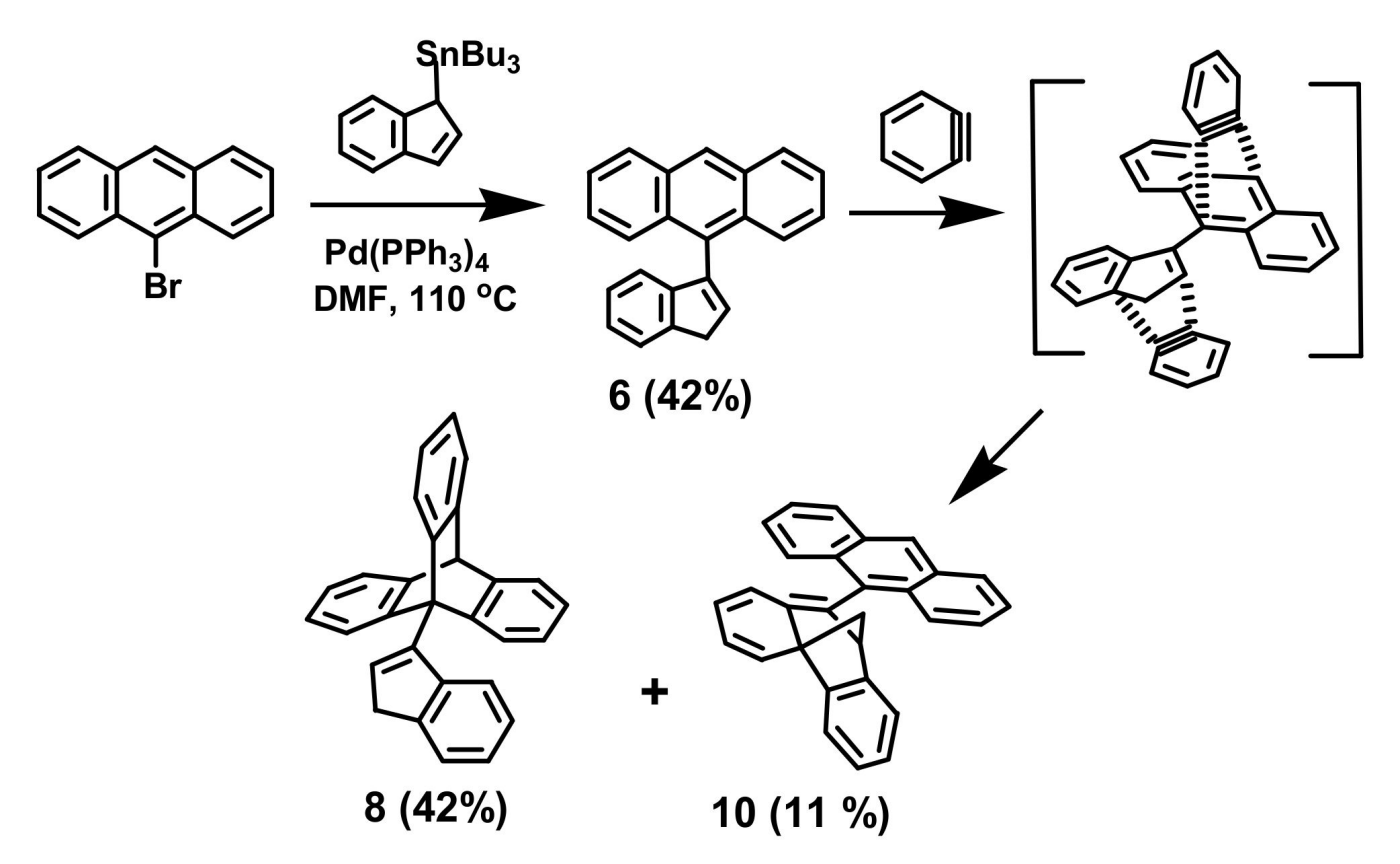
Palladium-catalysed route to **6**, and its benzyne adducts, **8** and **10**.

**Figure 5:**
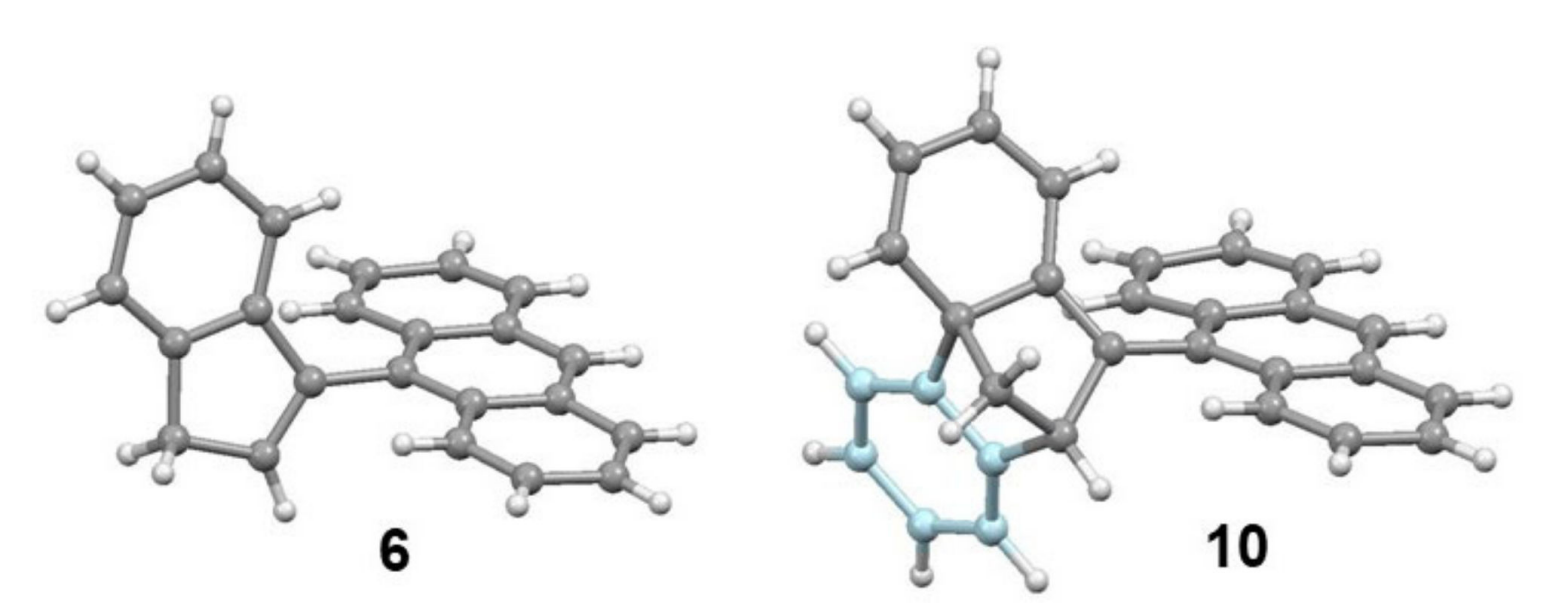
Molecular structures of 9-(3-indenyl)anthracene, **6**, and 10-(anthracen-9′-yl)-[4a,9]-methano-4a,9-dihydrophenanthrene, **10**.

**Figure 6:**
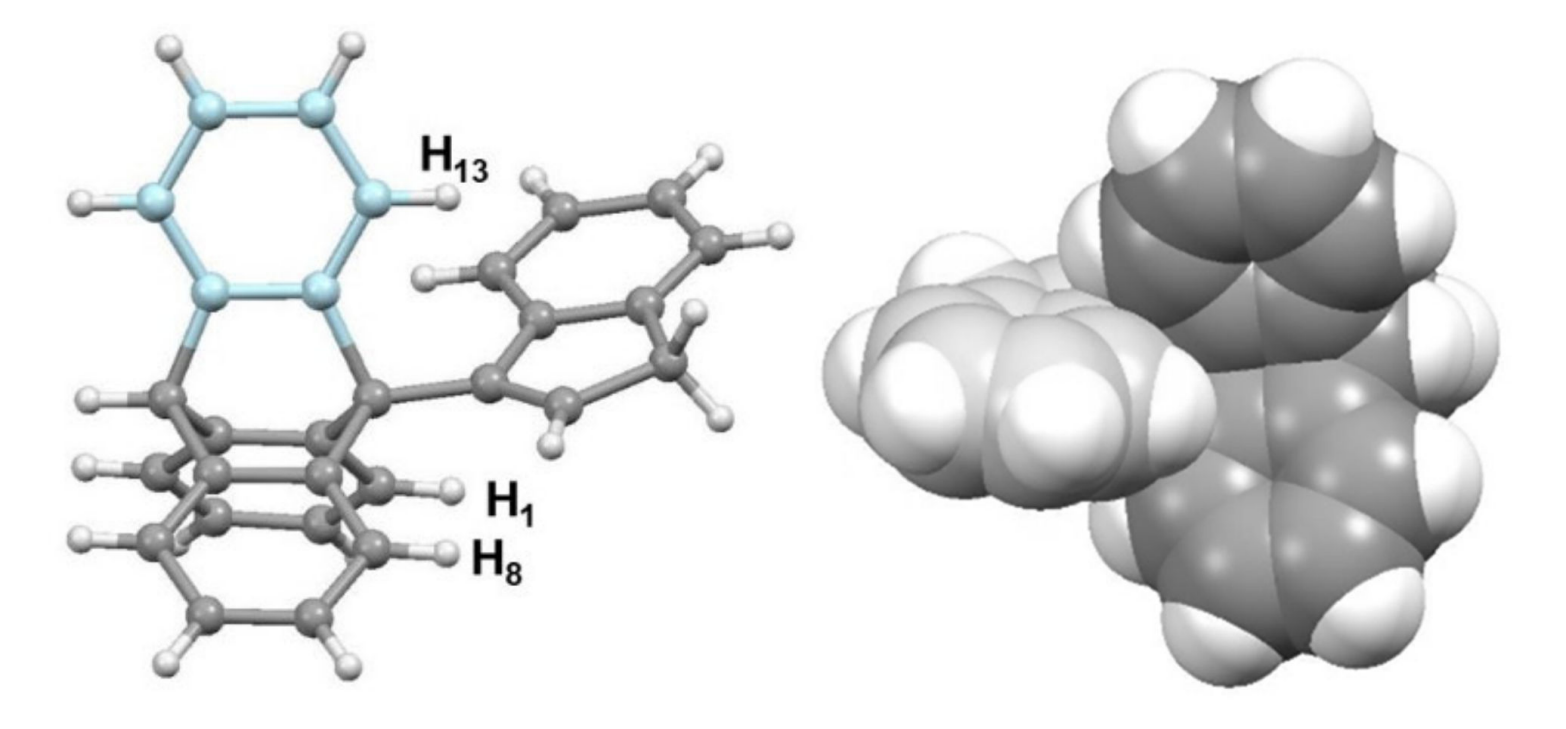
Molecular structure of 9-(3-indenyl)triptycene, **8**, and a space-fill view, from the reverse angle, to emphasise its molecular crowding.

Our initial target, 9-(3-indenyl)anthracene, 6, was successfully obtained in 42% yield via the Stille coupling of 9-bromoanthracene and 1-(tributylstannyl)indene catalysed by Pd(PPh_3_)_4_ in DMF [10]. Interestingly, benzyne addition to 6 occurred not only at the C(9) and C(10) positions of the anthracene to give the desired triptycene, 8, but also to the indenyl substituent, thus furnishing the [4 + 2] cyclo-adduct, 10 (Scheme 2). These three structures were validated by X-ray crystallography [10,11], and are shown in Figure 5 and Figure 6. These data revealed the extent of steric crowding between the indenyl and the peri-hydrogens at C(1), C(8) and C(13) of the triptycyl unit in 8 that engenders a 50 kJ mol^−1^ rotational barrier, as measured by variable-temperature NMR, even before incorporation of the organometallic moiety.

**Figure 7:**
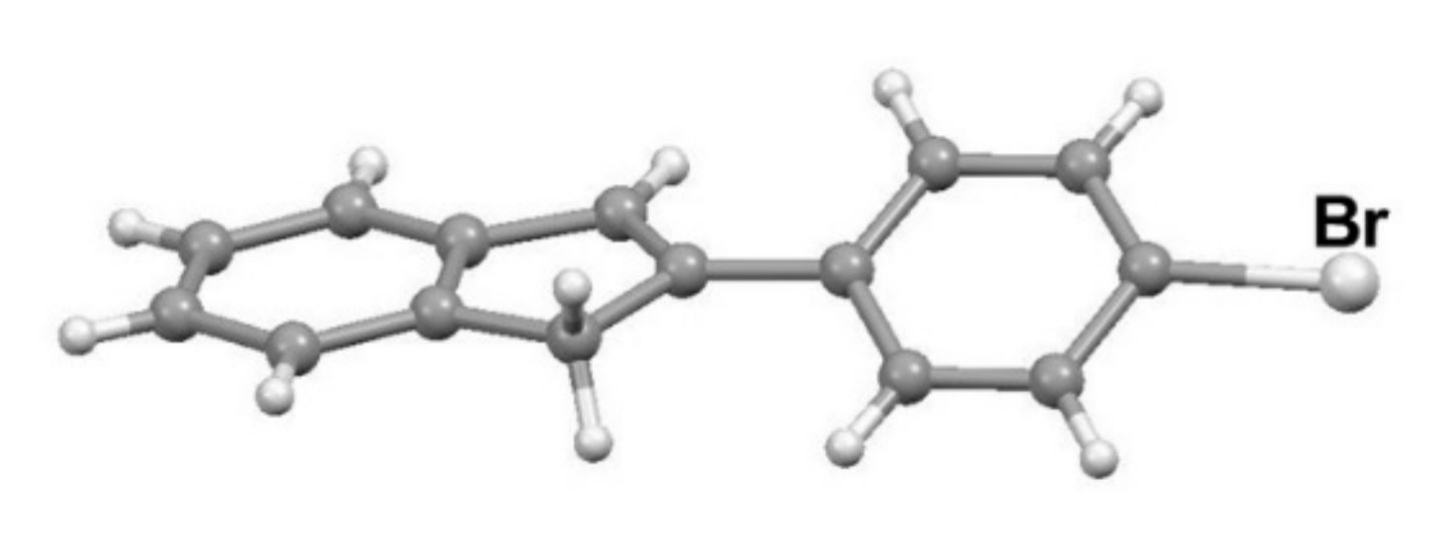
Molecular structure of 2-(4-bromophenyl)indene, **11**.

In light of these observations, it was decided to focus on the preparation of 9-(2-indenyl)anthracene, 7, the precursor to 9-(2-indenyl)triptycene. A previous report indicated that Heck-type palladium cross-couplings of indene with iodoarenes led primarily to formation of 2-arylindenes and, to a lesser extent, 3-arylindenes [12]. However, since their characterisations were based only on NMR data, we chose to perform the reaction of indene with 1-bromo-4-iodobenzene, using palladium acetate as the catalyst and triethylamine as the base [11]. Gratifyingly, the major product was identified unequivocally by X-ray crystallography as 2-(4-bromophenyl)indene, 11, whose structure appears as Figure 7.

**Scheme 3:**
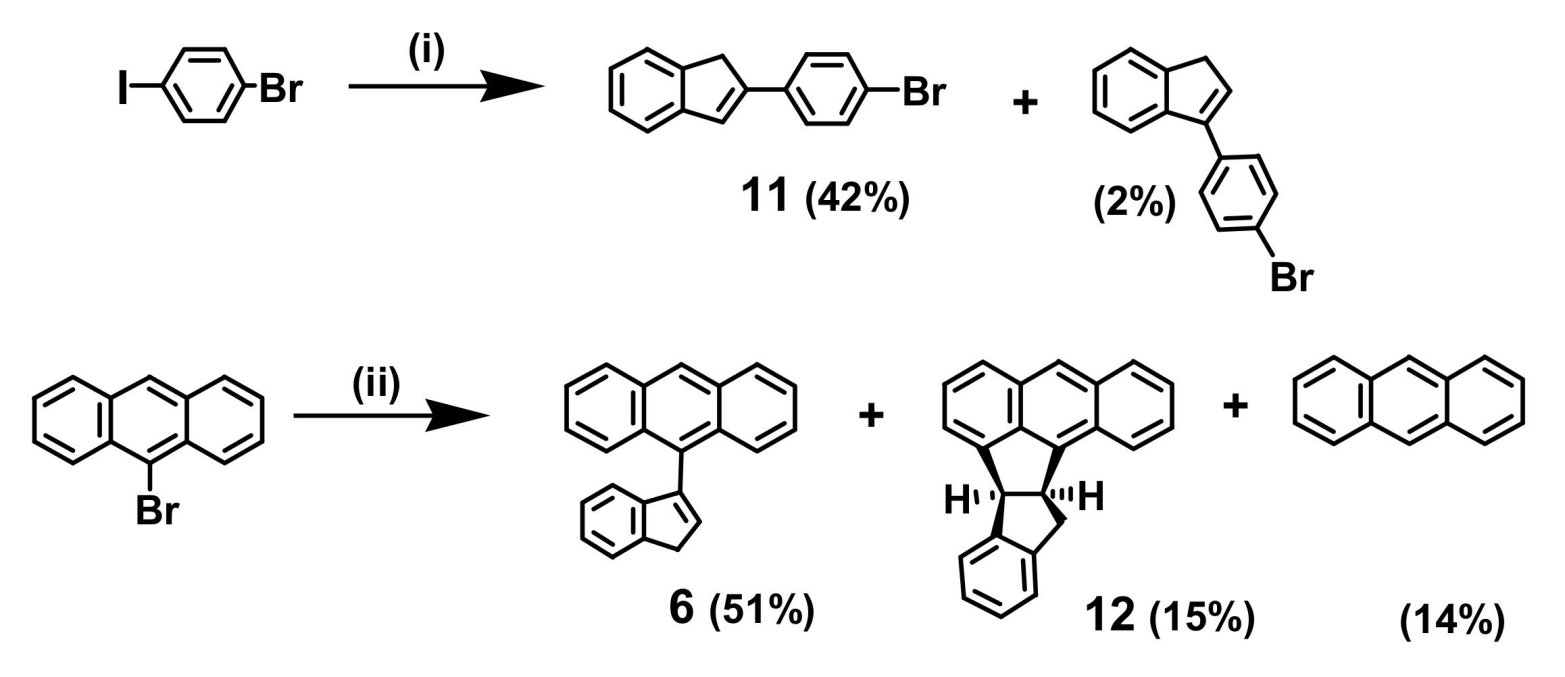
Pd-catalysed formation of arylindenes. Reagents and conditions: (**i**) indene, Pd(OAc)_2_, Et_3_N, 100 °C; (**ii**) as for (**i**) but using (*O*-tolyl_3_P)_2_PdCl_2_.

**Figure 8:**
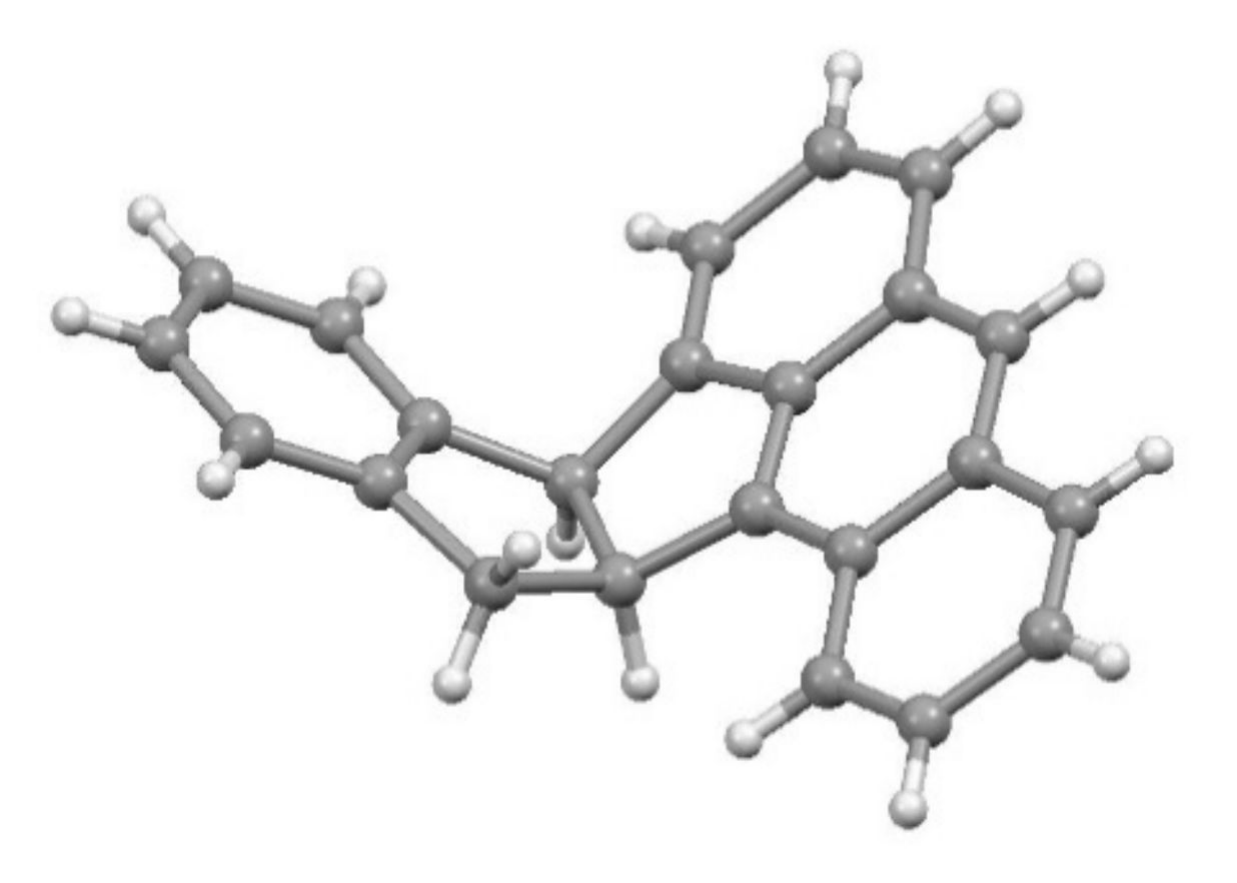
Molecular structure of indeno [1,2-*α*]-10,16-dihydroaceanthrylene, **12**.

As anticipated, the iodo substituent, rather than bromo, was replaced (Scheme 3, upper). Nevertheless, we found that bromobenzene and indene in the presence of dichloro-bis(tri-*O*-tolylphosphine)palladium(II) in DMF at 100 °C delivered 2-phenylindene in 90% yield. Therefore, with some confidence (unjustified, as it turned out), we carried out the reaction of indene with 9-bromoanthracene under these same conditions in the expectation of forming the desired 9-(2-indenyl)anthracene, 7, as the major isomer. However, as depicted in Scheme 3 (lower), the already known 9-(3-indenyl)anthracene, 6, together with the indeno-dihydroaceanthrylene, 12, (Figure 8) were the only cross-coupled products [11].

**Scheme 4:**
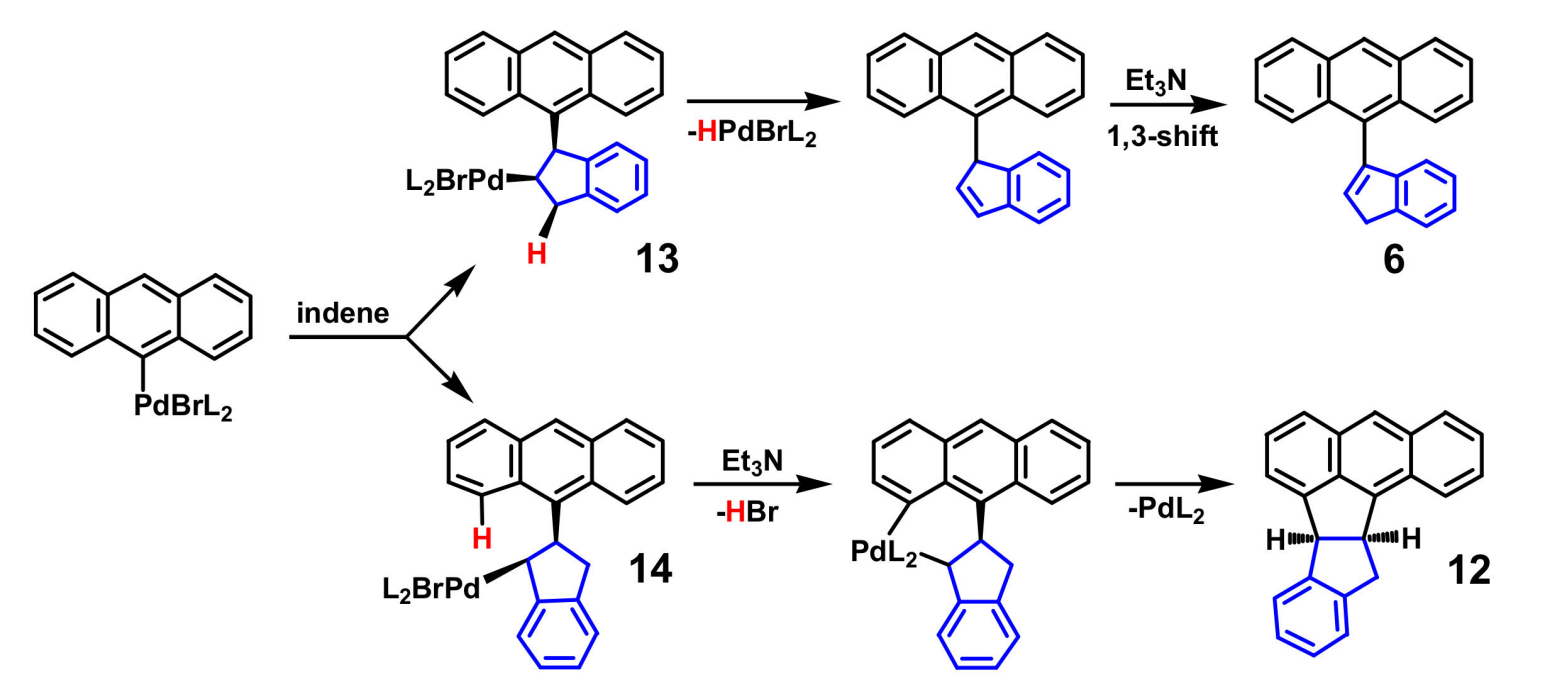
Proposed mechanisms for the indenylation of 9-bromoanthracene (L = PAr_3_).

These results may be rationalised in terms of the two different modes of insertion of indene into the palladium-aryl linkage of (9-anthracenyl)Pd(*O*-tolyl_3_P)_2_Br, thereby generating intermediates 13 and 14 (Scheme 4). In the former case, *syn*-elimination of HPd(*O*-tolyl_3_P)_2_Br occurs readily to yield 9-(1-indenyl)anthracene that subsequently rearranges to its 9-(3-indenyl)anthracene counterpart, 6. However, intermediate 14 lacks a suitably positioned hydrogen *syn* to palladium and so instead undergoes intramolecular palladation of the adjacent anthracene ring to form the aceanthrylene, 12. Such a mechanism should leave the two bridgehead hydrogens in a *syn* arrangement, as is indeed evident in the X-ray crystal structure shown in Figure 8, which emphasizes the folded nature of the molecule about the common bond linking the two five-membered rings.

**Scheme 5:**
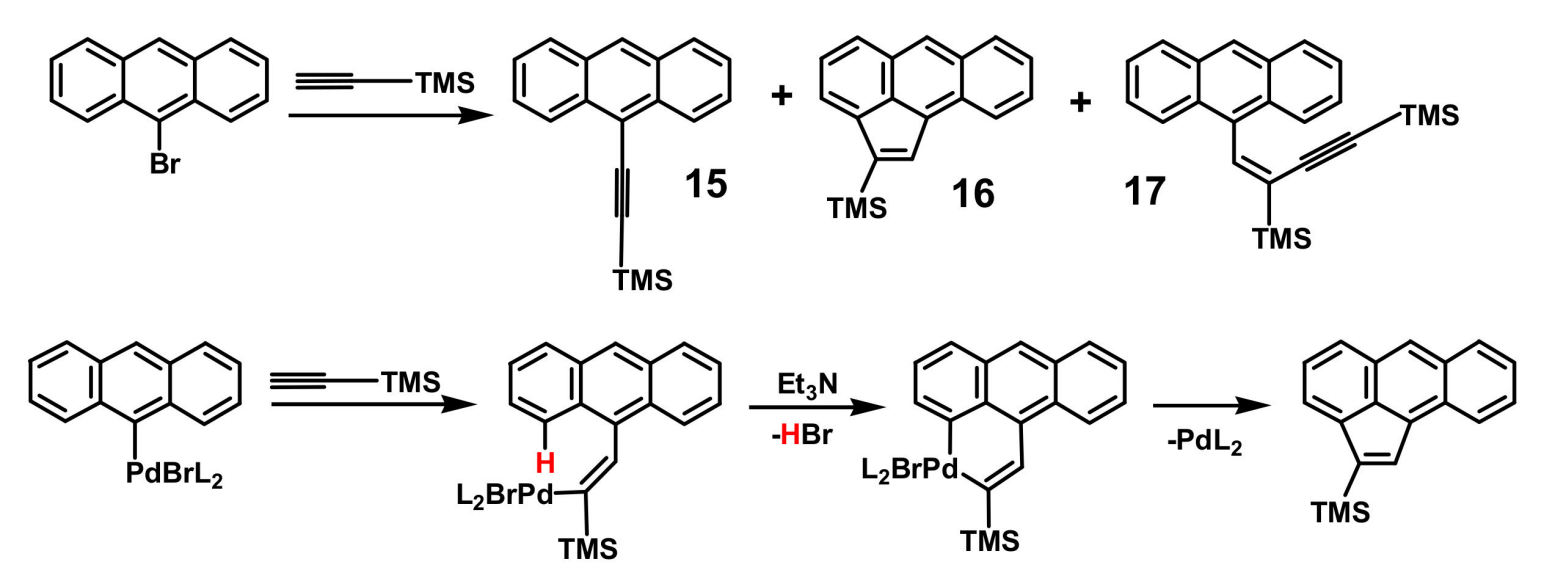
Formation of multiple products from the palladium-catalysed reaction of 9-bromoanthracene and ethynyltrimethylsilane.

We note that such a cyclopentenylation to form an aceanthrylene was first observed by Dang and Garcia-Garibay [13]. They found, surprisingly, that their attempt to prepare 9-(trimethylsilylethynyl)anthracene, 15, via the Sonogashira reaction between 9-bromoanthracene and ethynyltrimethylsilane in the presence of (Ph_3_P)_2_PdCl_2_, CuSO_4_/Al_2_O_3_ and Et_3_N in refluxing benzene gave instead 2-(trimethylsilyl)aceanthrylene, 16, as the major product. Once again, it is now evident that the lack of a *syn*-disposed hydrogen led to intramolecular palladation, as illustrated in Scheme 5. Since that time, Plunkett has elegantly exploited this cyclopentenylation process to produce polycyclic systems that represent fragments of fullerenes [14,15]. We note in passing that, depending on the particular Sonogashira conditions selected, the yield of 9-(trimethylsilylethynyl) anthracene, 15, has not only been improved to 98% [16], but also that one can produce 4-(9-anthracenyl)-1,3-bis(trimethylsilyl)-but-3-en-1-yne, 17, in moderate yields; a detailed mechanism has been advanced [17,18].

**Scheme 6:**
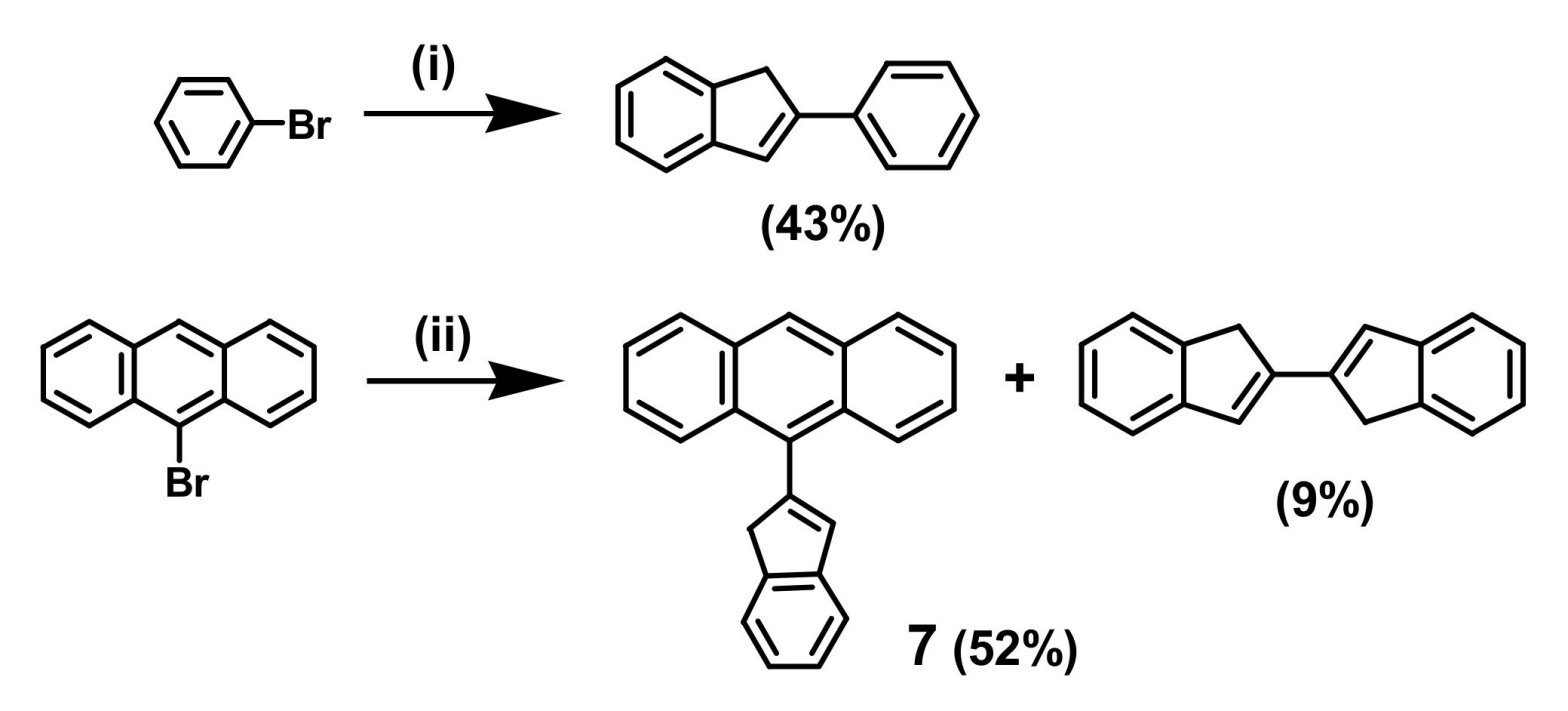
Pd-catalysed Suzuki-type coupling of aryl bromides. Reagents and conditions: (**i**) 2-indenylboronic acid, (Ph_3_P)_2_PdCl_2_, (1 mol%), ethanol-toluene, Na_2_CO_3_, 30 h, 75 °C; (**ii**) as for (**i**) but using (dppf)PdCl_2_.

Having now established that the Heck-type reaction of indene with 9-bromoanthracene provides an improved route to 9-(3-indenyl)anthracene, 6, rather than its desired 2-indenyl counterpart, 7, we chose instead to attempt a Suzuki-type cross-coupling, as illustrated in Scheme 6. The reaction of 9-bromoanthracene with 2-indenylboronic acid catalysed by dichloro-bis(diphenyl-phosphinoferrocene)palladium(II), (dppf)PdCl_2_, in ethanol-toluene at 75 °C, using Na_2_CO_3_ as the base, delivered 9-(2-indenyl)anthracene, 7, in 52% yield, together with the homo-coupled product, 2,2′-biindenyl (9%) [11].

**Scheme 7:**
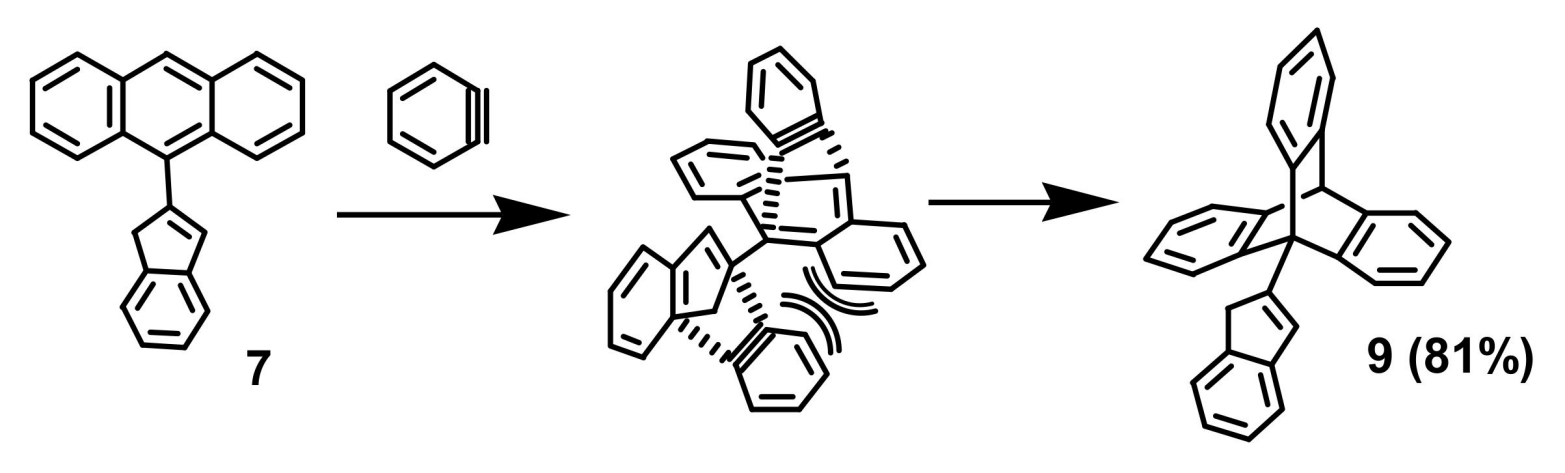
Reaction of benzyne and 9-2(indenyl)anthracene yields only a single adduct, **9**.

With 9-(2-indenyl)anthracene in hand, Diels-Alder addition of benzyne to form 9-(2-indenyl)triptycene, 9, proceeded in 81% yield. This much improved yield of 9 compared to that of 9-(3-indenyl)triptycene, 8 (42%) may be attributed to the trajectory of approach for a potential [4 + 2] cycloaddition of benzyne to the five-membered ring of either 6 or 7. As depicted in Scheme 2 and Scheme 7, in the former case, access to both the anthracene and the indene is available, whereas, in the 2-indenyl system, the latter process would be blocked by the proximity of the planar anthracenyl, thus leading to a single product in enhanced yield [11].

### 2.2. Rotational Barriers in di-Indenyl Anthracenes

**Scheme 8:**
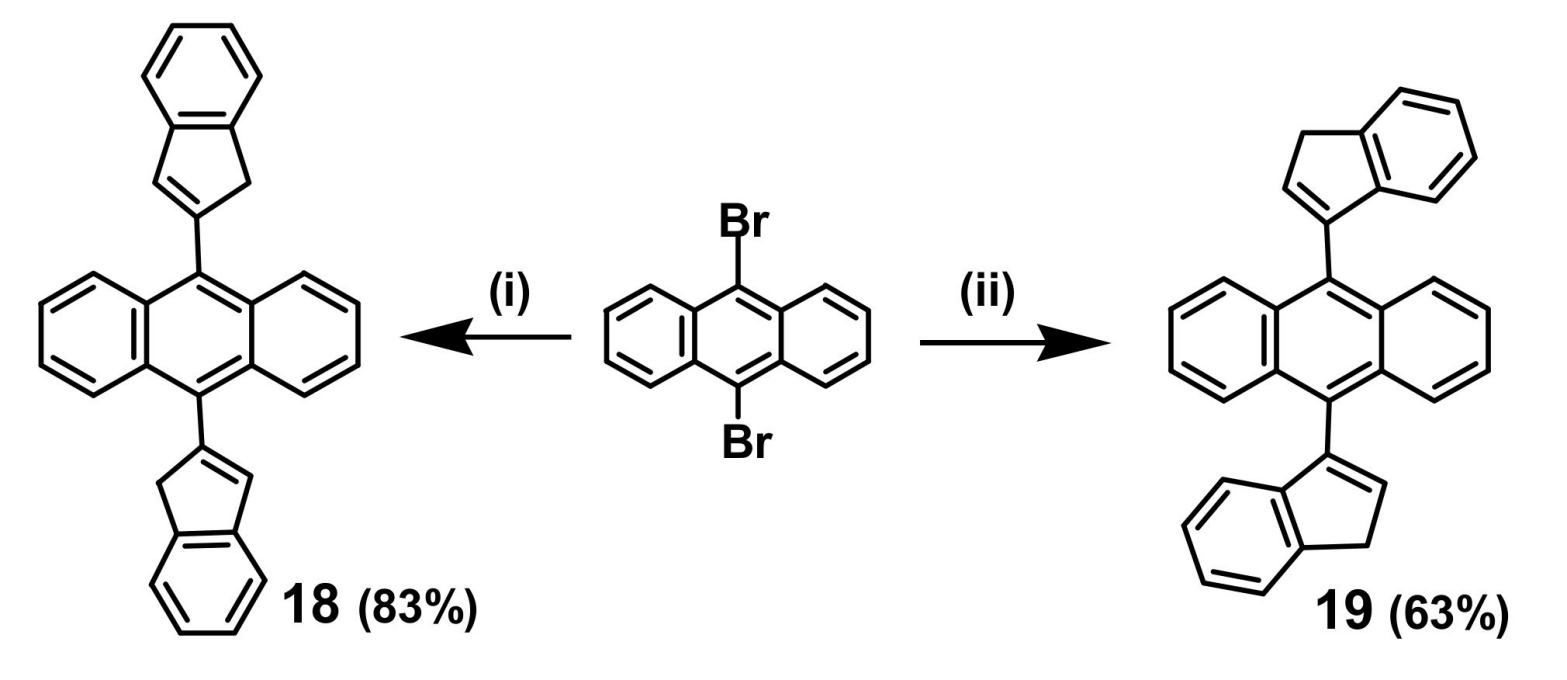
Pd-catalysed formation of di-indenylanthracenes, **18** and **19**. Reagents and conditions: (**i**) 2-indenylboronic acid, (dppf)PdCl_2_, Na_2_CO_3_, ethanol-toluene, 80 h, 80 °C; (**ii**) 1-(trimethylstannyl)indene, (O-tol_3_P)_2_PdCl_2_, 1,4-dioxane, 40 h, 120 °C.

**Figure 9:**
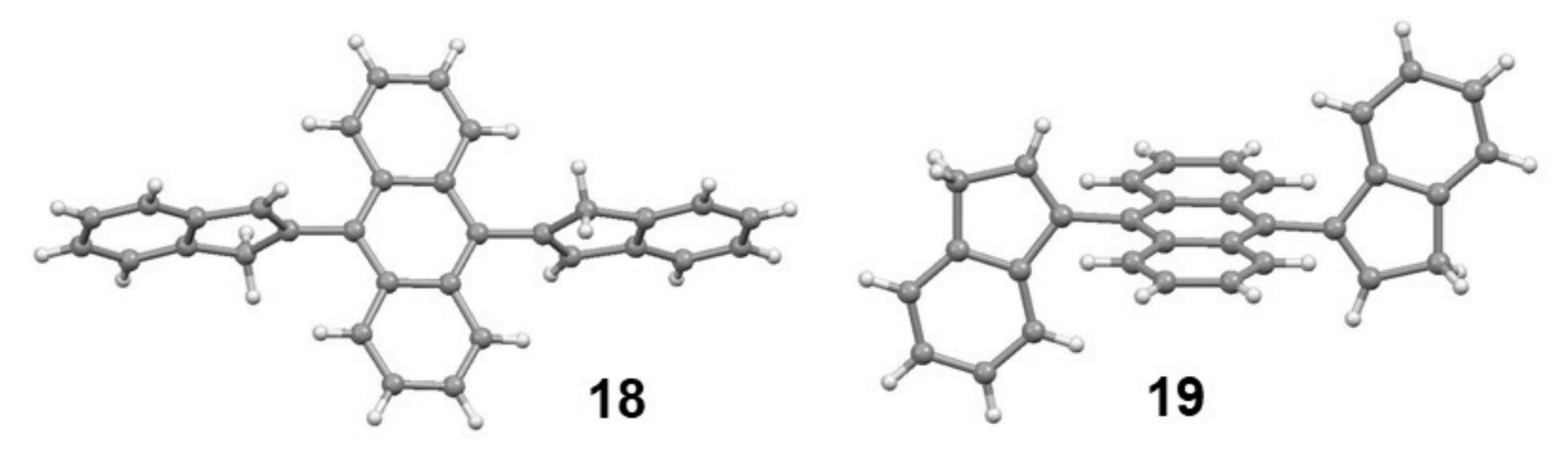
Structures of 9,10-di(2-indenyl)anthracene, **18**, and *anti*-9,10-di(3-indenylanthracene, **19**.

Having established convenient palladium-catalysed routes to 9-(3-indenyl)anthracene, 6, and 9-(2-indenyl)anthracene, 7, this work was then extended to include their di-indenyl counterparts, starting from 9,10-dibromoanthracene (Scheme 8). Thus, 9,10-di(2-indenyl)anthracene, 18, was prepared in 83% yield via Suzuki cross-coupling with 2-indenylboronic acid mediated by (dppf)PdCl_2_ in ethanol-toluene. The attempted Heck-type route to 9,10-di(3-indenyl)anthracene led predominantly to formation of dihydroaceanthrylenes, but the Stille reaction of 1-(trimethylstannyl)indene, 9,10-dibromoanthracene and dichloro-bis(tri-*O*-tolylphosphine)-palladium(II) in 1,4-dioxane gave the desired product, 19, in 63% yield. The X-ray crystal structures of 18 and 19 appear in Figure 9, and exhibit interplanar indenyl-anthracenyl dihedral angles of 81.7° and 81.5°, respectively [19].

It was apparent that 9,10-di(3-indenyl)anthracene, 19, exists as an almost 50/50 mixture of *syn* and *anti* atropisomers that, fortunately, are separable by column chromatography. Their barrier to interconversion was evaluated in a kinetic experiment monitored by NMR as ca. 105 kJ mol^−1^, approximately twice that found in 9-(3-indenyl)triptycene. Likewise, 9,10-di(2-indenyl)anthracene, 18, exists as an *syn*/*anti* mixture; these atropisomers are not separable but their interconversion can be monitored by V-T ^13^C NMR that yielded a rotational barrier of ca. 55 kJ mol^−1^. At first sight, it may be surprising that the 3-indenyl rotation barrier in the anthracene, 19, is so much greater than in the corresponding triptycene, 8. However, simulated virtual rotation in 8 reveals that, as the benzo ring of the 3-indenyl unit passes a triptycyl blade, the five-membered ring can bend into the space between the other two blades, thus minimising the energy cost of the rotation. This “duck and dodge” mechanism is not available in the 3-indenylanthracenes in which two unfavourable coplanar H•••H contacts are attained simultaneously [19].

### 2.3. Cycloadditions of Benzyne to 2-Phenylindene

**Scheme 9:**
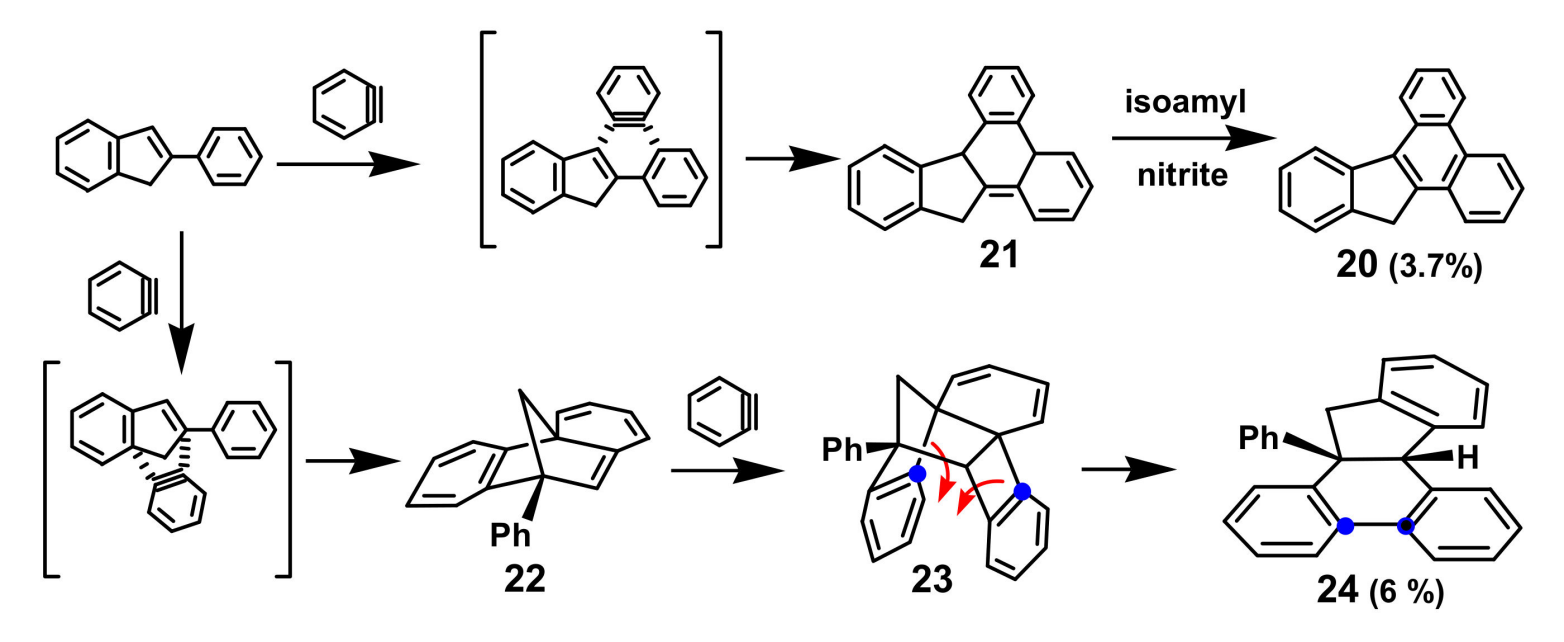
Cycloaddition reactions of benzyne to 2-phenylindene.

Intrigued by the unexpected cycloaddition of benzyne to the indenyl substituent of 9-(3-indenyl)anthracene to form the methano-dihydrophenanthrene, 10, we chose to investigate the reaction of benzyne with 2-phenylindene. Although the majority of the starting indene was recovered, two products were isolated and fully characterised as benzyne adducts [11]. The first was readily identified spectroscopically as the known indeno-phenanthrene, 20, resulting from the addition of a single benzyne to 2-phenylindene. Presumably, the initially formed dihydrophenanthrene, 21, was further oxidised in the presence of excess isoamyl nitrite (Scheme 9).

**Figure 10:**
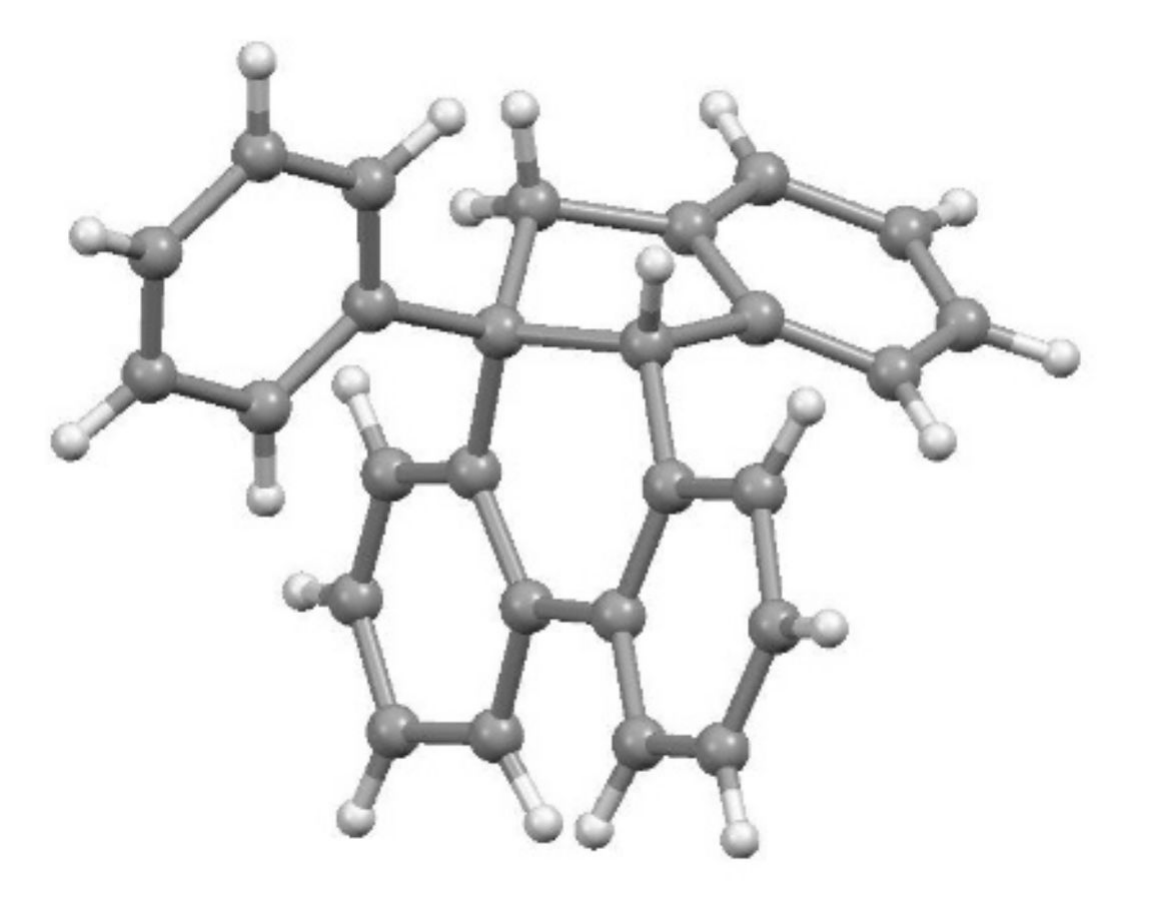
Structure of 13a-phenyl-13,13a-dihydro-8b*H*-indeno-[1,2-*l*]-phenanthrene, **24**.

The formula of the second product corresponded to the addition of two benzyne units to 2-phenylindene; one can envisage the first step as the [4 + 2] cycloaddition to the indenyl moiety to form 22, entirely analogous to the reaction of benzyne with 9-(3-indenyl)anthracene to form 10. In the second step, [2 + 2] addition yields a cyclobutene, 23, that adopts the *syn*, rather than the *anti*, configuration because of the presence of the adjacent phenyl substituent. Subsequent thermolysis can open up the four-membered ring and bring about rearrangement to the observed product 24 that was unequivocally characterised by X-ray crystallography (Figure 10). The thermodynamic driving force for such a process would be the relief of steric strain in the cyclobutene ring, and the recovery of aromatic character in the original six-membered ring of the indene [11].

### 2.4. Organometallic Derivatives of Indenyl Anthracenes

The palladium-mediated ready availability of 2-phenylindene, 9-(2-indenyl)anthracene and 9-(3-indenyl)anthracene, as well as their corresponding triptycenes, prompted an investigation of the syntheses, structures and dynamic behaviour of some of their organometallic derivatives.

**Scheme 10:**
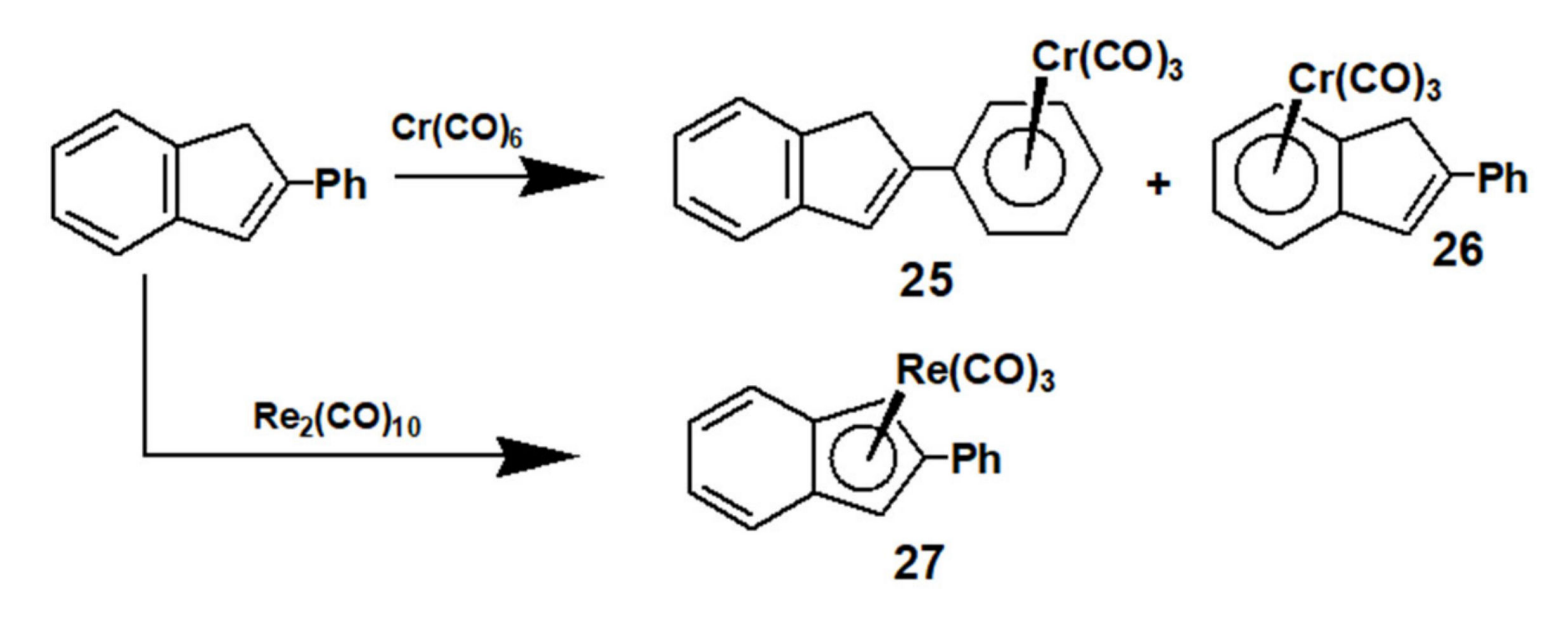
Chromium and rhenium tricarbonyl complexes of 2-phenylindene.

**Figure 11:**
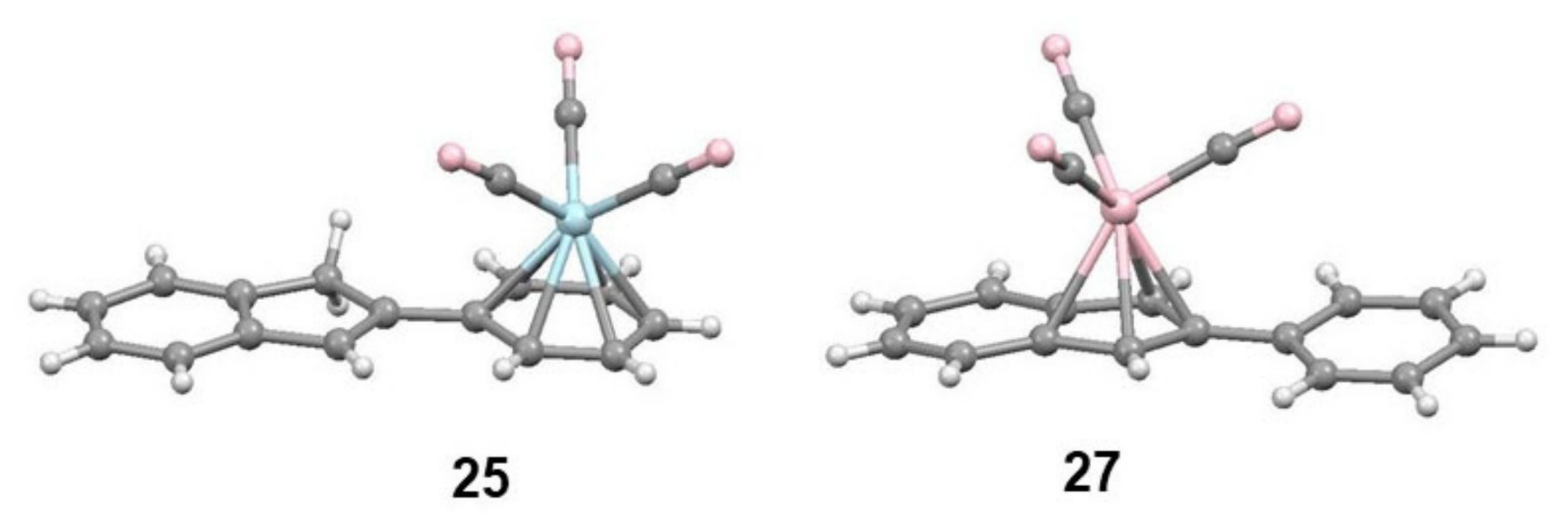
Molecular structures of [η^6^-(2-indenyl)benzene]tricarbonylchromium, **25**, and (η^5^-2-phenylindenyl)tricarbonylrhenium, **27**.

The 2-phenylindene reacts with Cr(CO)_6_ in 1,4-dioxane at 125 °C to form two isomeric complexes wherein the Cr(CO)_3_ tripod is attached in an η^6^ fashion either to the phenyl substituent, as in 25, or to the six-membered ring of the indene, as in 26 (Scheme 10). (η^5^-2-phenylindenyl)Re(CO)_3_, 27, was prepared either directly by heating the ligand with Re_2_(CO)_10_ in decalin in a sealed tube at 160 °C or indirectly by transmetallation of 2-phenyl-1-trimethylstannylindene. The structures of 25 and 27 appear as Figure 11 and show that the indenyl and phenyl planes differ only slightly from coplanarity [20].

We note that complexes 25 and 27 can each adopt a mirror-symmetric (*C*_s_) conformation; in the chromium case, the indenyl unit must be aligned orthogonal to the plane of the phenyl ring (dihedral angle of 90°), whereas, in the rhenium complex, 27, the molecular mirror plane only bisects both the indenyl and phenyl rings when they are coplanar (dihedral angle of 0°). In all these cases, the rotation barrier about the axis connecting the rings is very low; DFT calculations suggest values in the range of 20–30 kJ mol^−1^ [20].

**Scheme 11:**
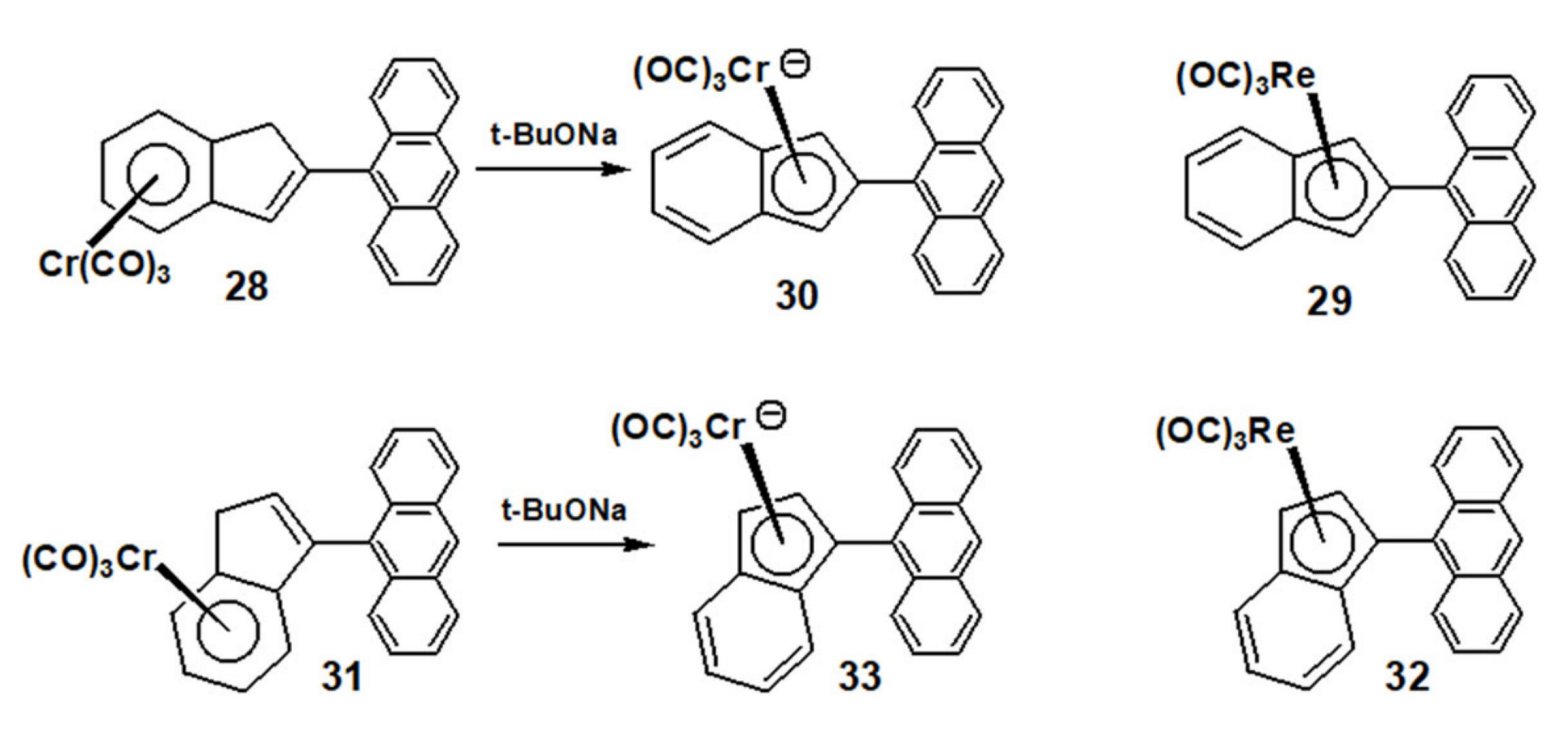
Cr and Re carbonyl complexes of 2-indenyl- and 3-indenyl-anthracene.

**Figure 12:**
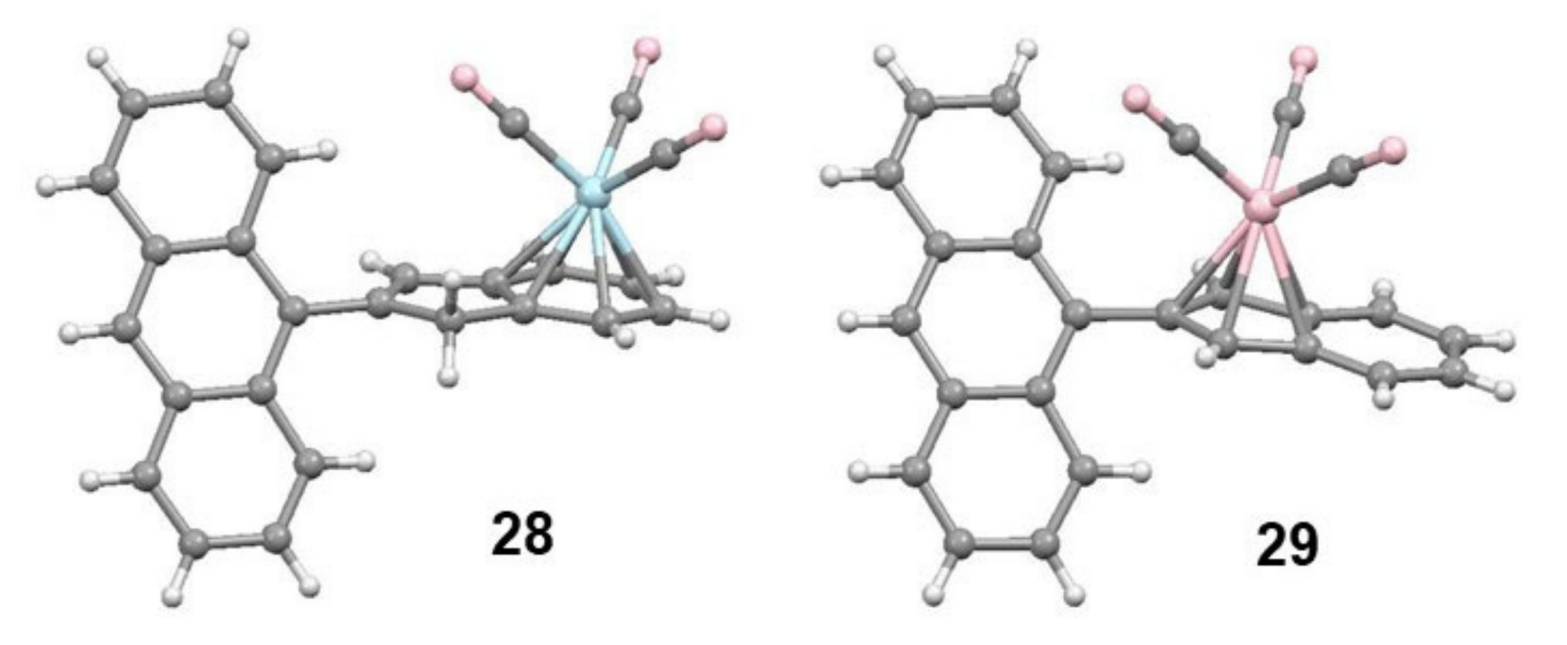
Molecular structures of [η^6^-2-(9-anthracenyl)indene]tricarbonylchromium, **28**, and [η^5^-2-(9-anthracenyl)indenyl]tricarbonylrhenium, **29**.

As shown in Scheme 11, the syntheses of the chromium and rhenium tricarbonyl complexes of 9-(2-indenyl)anthracene, 28 and 29, respectively, parallel those of their 2-phenylindene counterparts. However, the considerably larger wingspan of the anthracenyl moiety brings about noticeable changes in their structures and dynamic behaviour; the interplanar indenyl anthracenyl angles in the chromium and rhenium complexes were found to be 62° and 52°, respectively (Figure 12).

In the η^6^-bonded chromium system, 28, the rotational barrier for equilibration of the benzo rings of the anthracene was measured by V-T NMR as ~63 kJ mol^−1^, a value 15 kJ mol^−1^ higher than that found for the (η^5^-indenyl)rhenium complex, 29, despite the Cr(CO)_3_ tripod being further away from the anthracene. However, one should recall that the NMR experiment yields a value for the energy separation between the ground state and the transition state for the dynamic process. In this case, rather than invoking a decrease in the energy of the transition state, it is more likely that the ground state has been raised by steric interactions with the methylene hydrogen atoms rather than with the metal carbonyl tripodal fragment, thus lowering the observed barrier. It is, therefore, particularly noteworthy that upon deprotonation of [η^6^-2-(9-anthracenyl)indene]tricarbonylchromium, 28, which brings about a haptotropic shift of the tripod to form the anion [η^5^-2-(9-anthracenyl)indenyl]tricarbonylchromium, 30, closely analogous to the rhenium complex, 29, this rotational barrier has once again decreased, an effect we designated as an “anti-braking” phenomenon.

**Figure 13:**
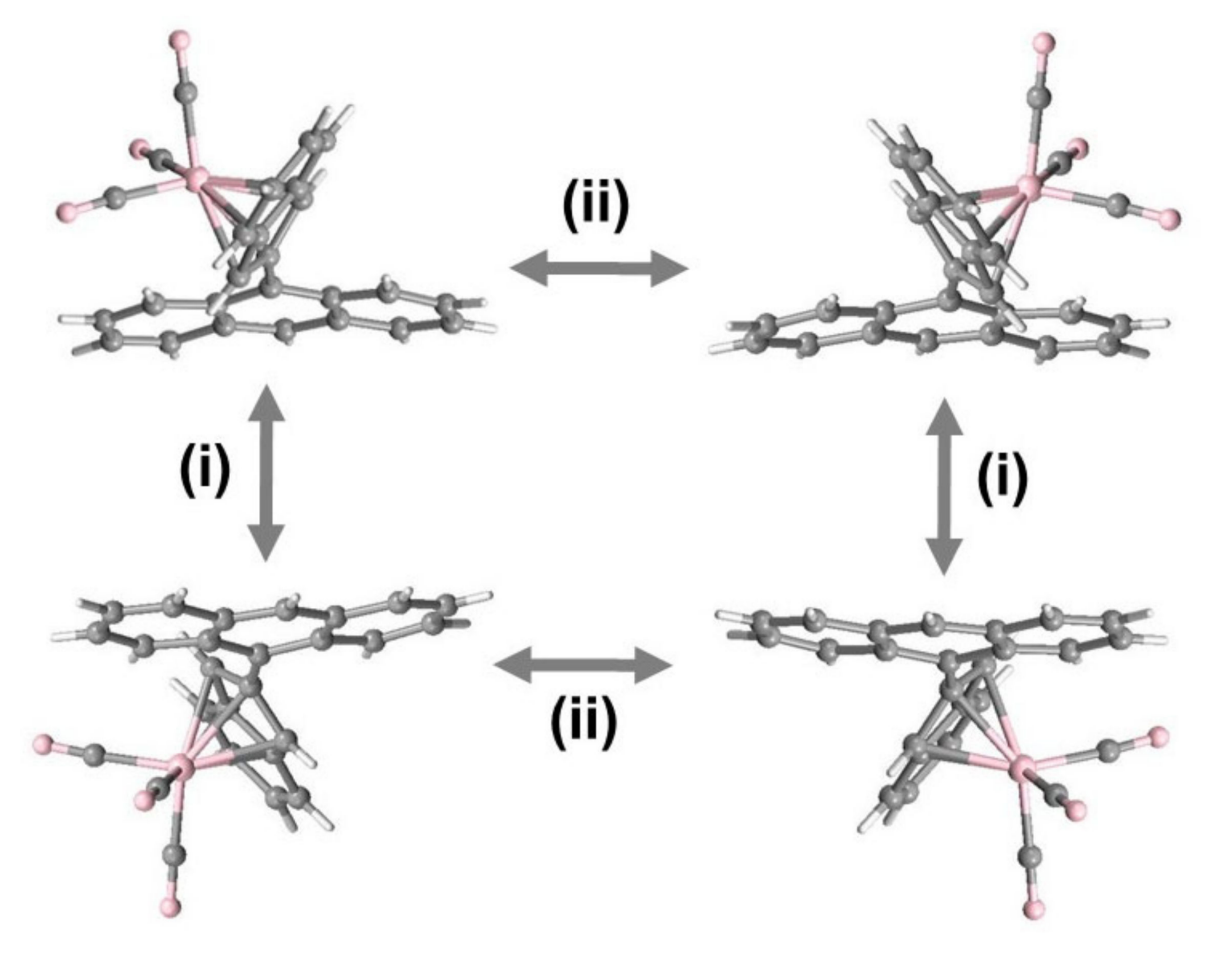
Exchange behaviour in the indenyl-anthracene rhenium complex **29**. (**i**) Low-energy (C_s_ symmetric) up/down oscillation of the indenyl-Re(CO)_3_ group relative to the anthracene ring plane; (**ii**) higher-energy process whereby side-to-side rotation of the indenyl-Re(CO)_3_ moiety about the C(9)-indenyl linkage equilibrates the terminal benzo rings.

As depicted in Figure 13, in 29, oscillation of the (indenyl)Re(CO)_3_ moiety across the mirror plane containing the anthracenyl framework, which equilibrates each of the three pairs of indenyl protons but maintains the inequivalence of the benzo rings of the anthracene, has a barrier too low to be accessed by V-T NMR even at −80 °C. In contrast, a window-wiper type of motion that equilibrates the terminal rings of the anthracene has to surmount a 48 kJ mol^−1^ barrier that arises because of the build-up of steric interactions as the indenyl and anthracenyl fragments approach coplanarity. Complete 360° rotation to generate effective time-averaged *C*_2v_ symmetry requires that both processes are operative [20]. 

**Figure 14:**
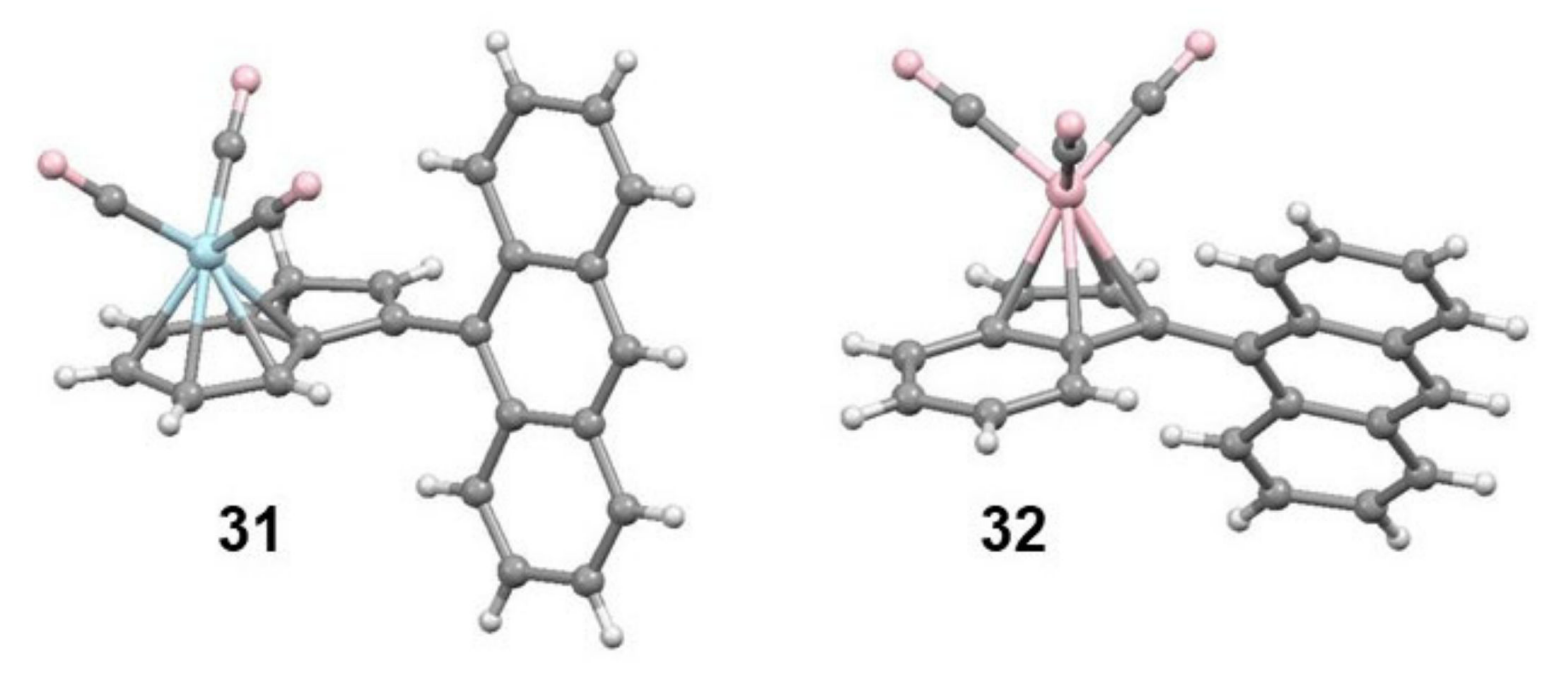
Molecular structures of [η^6^-3-(9-anthracenyl)indene]tricarbonylchromium, **31**, and [η^5^-3-(9-anthracenyl)indenyl]tricarbonylrhenium, **32**.

The analogous η^6^-chromium and η^5^-rhenium complexes of 9-(3-indenyl)anthracene, 31 and 32, respectively, have also been prepared and structurally characterised (Figure 14). In these molecules, the interplanar indenyl-anthracenyl dihedral angles are 75.5° and 56.5°, respectively. As previously discussed, in the free ligand steric interactions between the peri-hydrogens, H(1) and H(8), of the anthracene skeleton and the indenyl substituent give rise to a rotational barrier of ca. 105 kJ mol^−1^. Following the pattern seen with [η^5^-2-(9-anthracenyl)indenyl]tricarbonylrhenium, 29, the barrier in 32 was reduced to 90 kJ mol^−1^, while that for the chromium complex, 31, was somewhat higher at ~96 kJ mol^−1^. Furthermore, as shown in Scheme 11, deprotonation of 31 initiated the η^6^ to η^5^ haptotropic shift, forming anion 33, in which the rotational barrier had once again fallen to 90 kJ mol^−1^.

### 2.5. Organometallic Derivatives of Indenyl Triptycenes

**Scheme 12:**
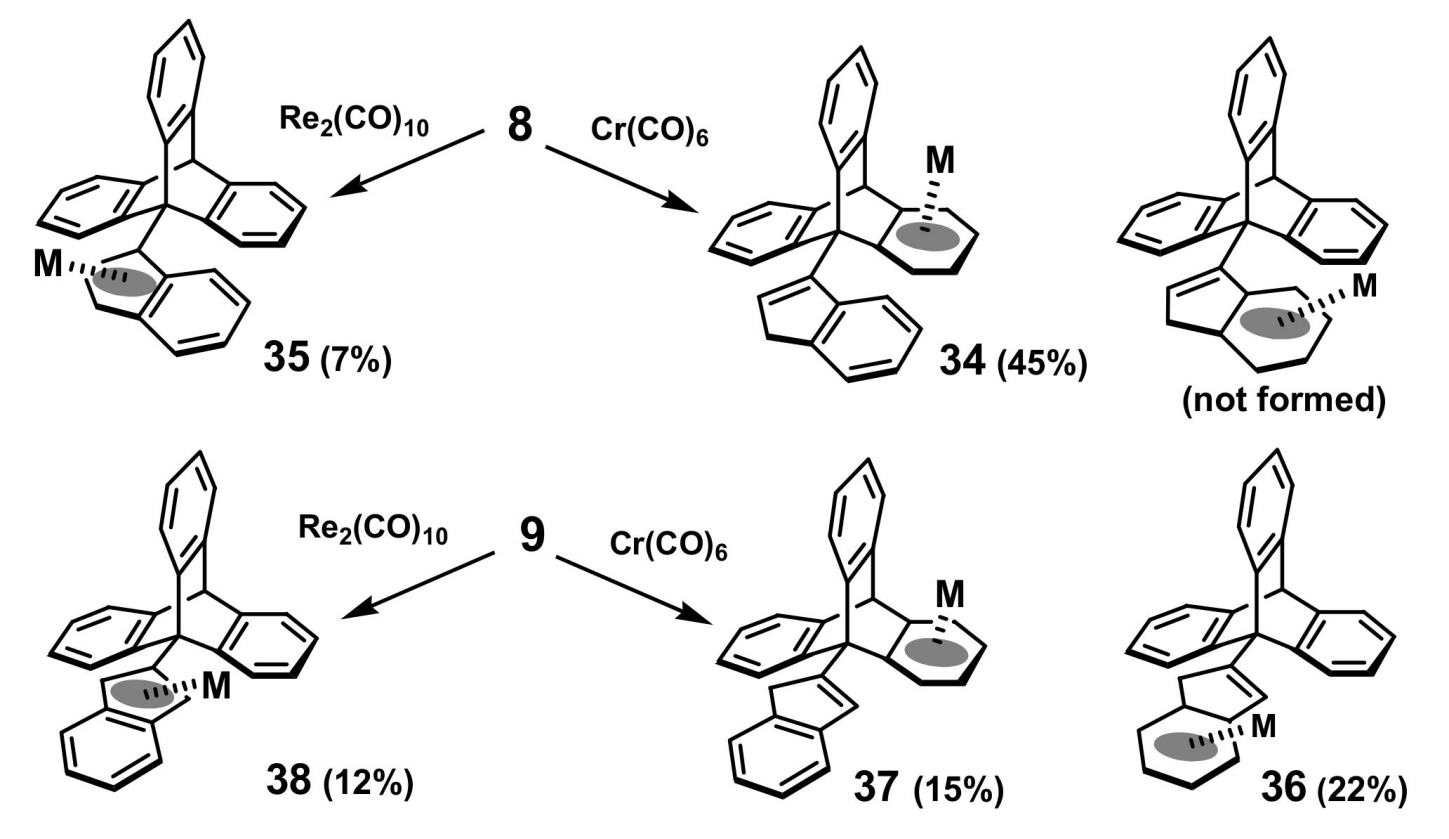
Cr and Re complexes of 9-(3-indenyl)triptycene and 9-(2-indenyl)triptycene.

**Figure 15:**
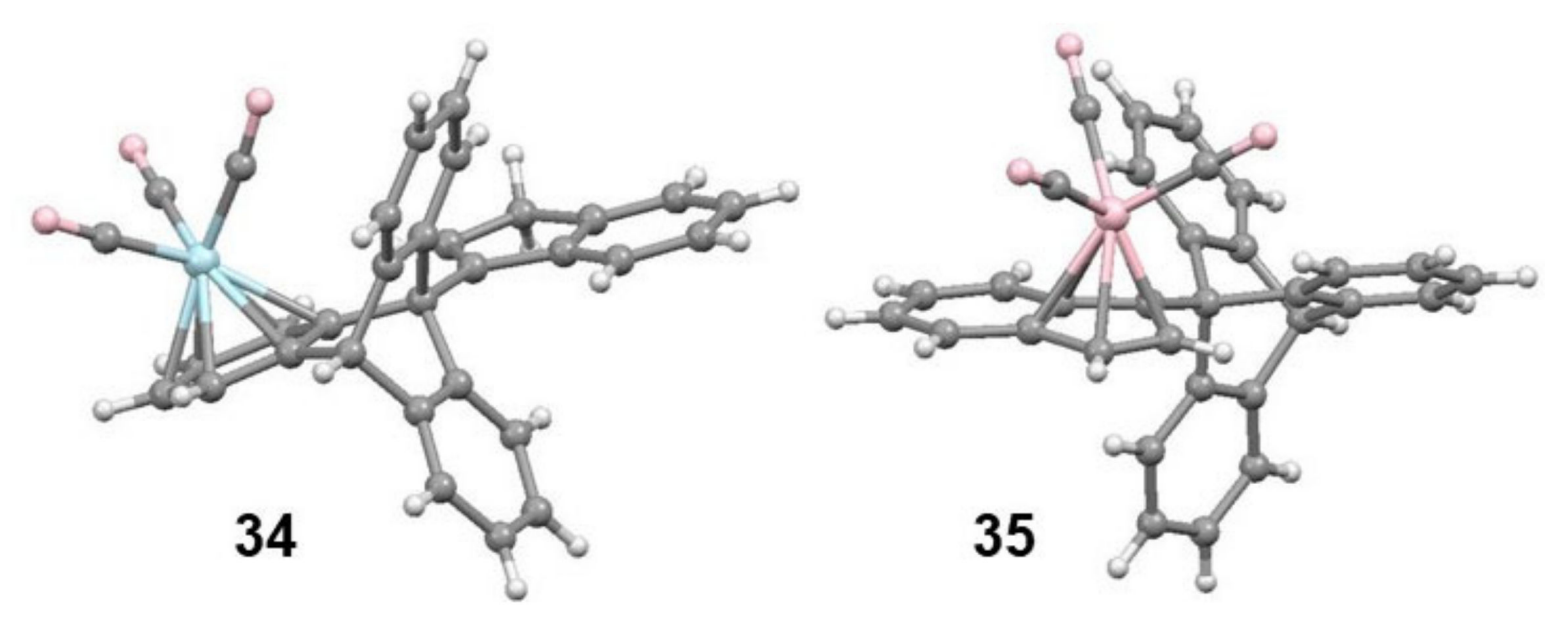
Structures of [η^6^-9-(3-indenyl)-1,2,3,4,4a,9a-triptycene]tricarbonyl-chromium, **34**, and [η^5^-3-(9-triptycyl)indenyl]tricarbonylrhenium, **35**.

Attempts to coordinate M(CO)_3_ tripods, where M = Cr, Mn or Re, to indenyl triptycenes led to a number of different products (Scheme 12). Dealing first with 9-(3-indenyl)triptycene, 8, reaction with Cr(CO)_6_ gave only a single isomer, 34, in which the tricarbonylchromium fragment is sited on a benzo ring of the triptycene (Figure 15), rather than on the six-membered ring of the indenyl substituent [10]. An alternative approach, addition of benzyne to 31, in which the Cr(CO)_3_ moiety is already in place, was unsuccessful. However, reaction of 8 with Re_2_(CO)_10_ gave a low yield of the η^5^-Re(CO)_3_ complex, 35 [20]. We note in particular that the indenyl ring in 35 is oriented such that the tricarbonylrhenium tripod is aligned with a valley between two blades (Figure 15).

**Figure 16:**
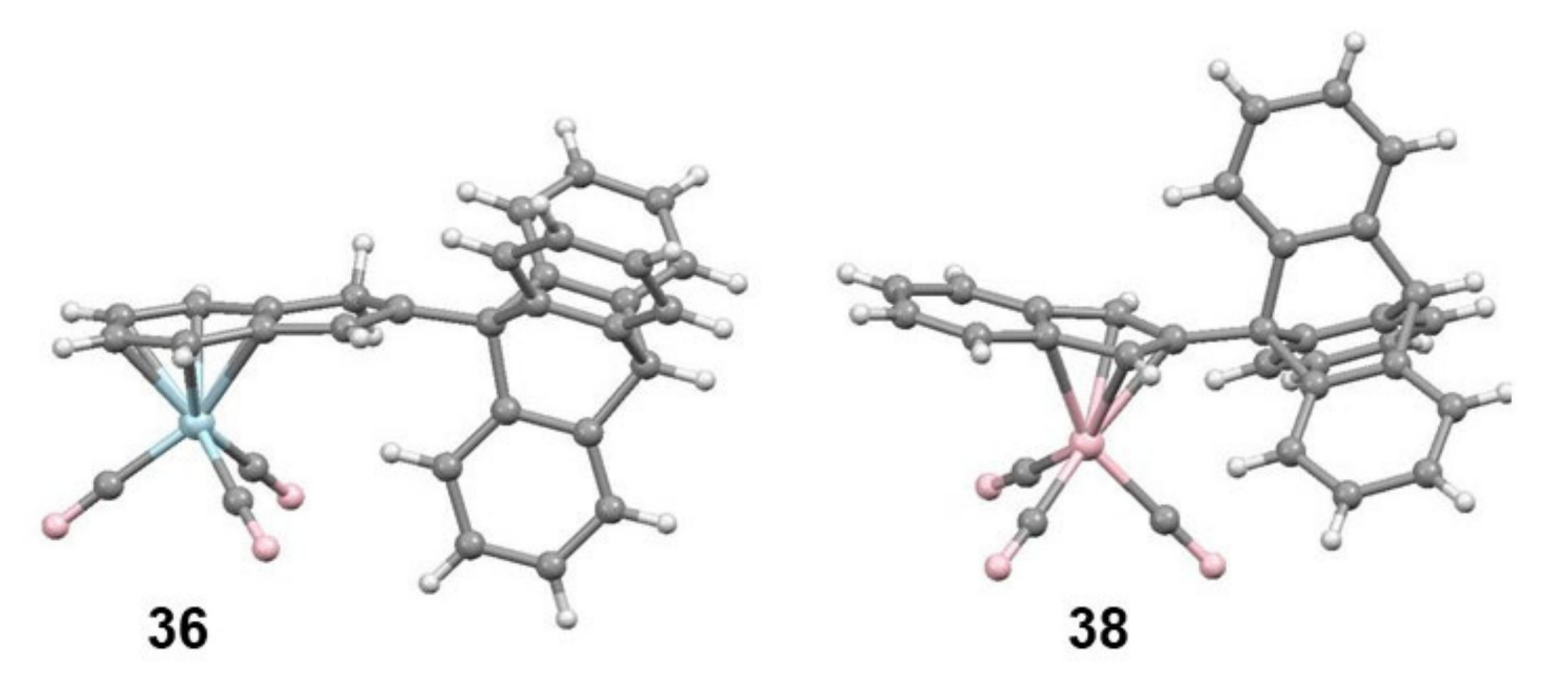
Structures of [η^6^-2-(9-triptycyl)indene]tricarbonylchromium, **36**, and [η^5^-2-(9-triptycyl)indenyl]tricarbonylrhenium, **38**.

The result of coordinating metal carbonyl tripods to 9-(2-indenyl)triptycene, 9, was more straightforward, and ultimately more satisfying [21]. As depicted in Scheme 12, the reaction with Cr(CO)_6_ furnishes two products, the target molecule, 36, in which the Cr(CO)_3_ unit is coordinated to the six-membered ring of the indene (Figure 16), and also its triptycyl blade complexed isomer, 37. Concomitantly, reaction with Re_2_(CO)_10_ delivered the η^5^-Re(CO)_3_ complex 38. The analogous (and isostructural) η^5^-Mn(CO)_3_ complex, 39, was also prepared, by reaction of the deprotonated ligand with BrMn(CO)_5_.

A ^13^C NMR study on the η^5^-rhenium complex 38 revealed that, at room temperature, the triptycyl resonances are each split into a 2:1 pattern, clearly indicating that paddlewheel rotation was slow on the NMR time-scale. However, upon raising the temperature, the gradual onset of line broadening, together with computer simulation of the spectra, yielded a barrier of 84 ± 2 kJ mol^−1^ for equilibration of the three benzo blades; the manganese analogue, 39, behaved similarly. In contrast, in the η^6^-chromium complex, 36, peak decoalescence even at low temperatures was never apparent, revealing that paddlewheel rotation continued essentially unhindered.

**Scheme 13:**
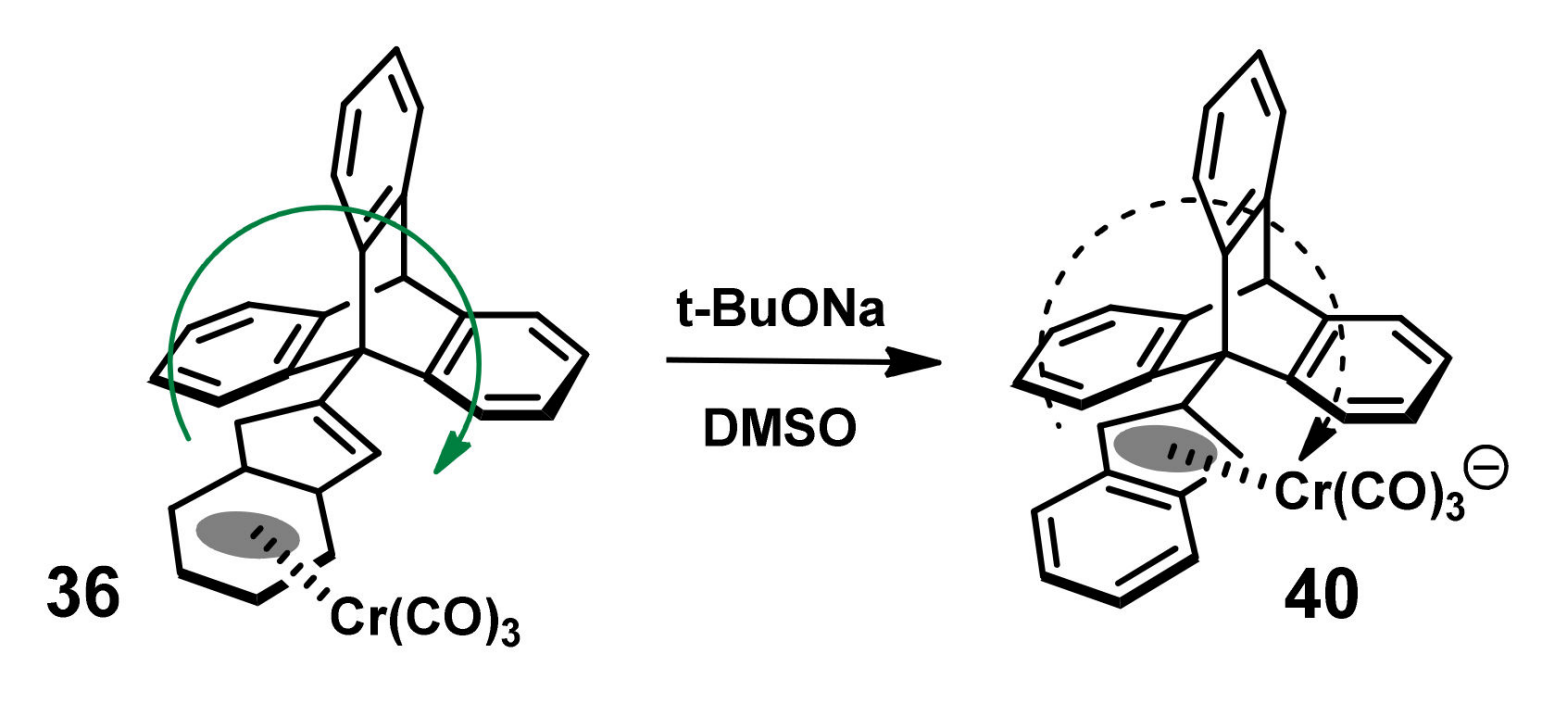
Deprotonation of **36** brings about an η^6^ → η^5^ haptotropic shift, forming **40**, in which paddlewheel rotation (dashed arrow) is dramatically slowed on the NMR time-scale.

**Figure 17:**
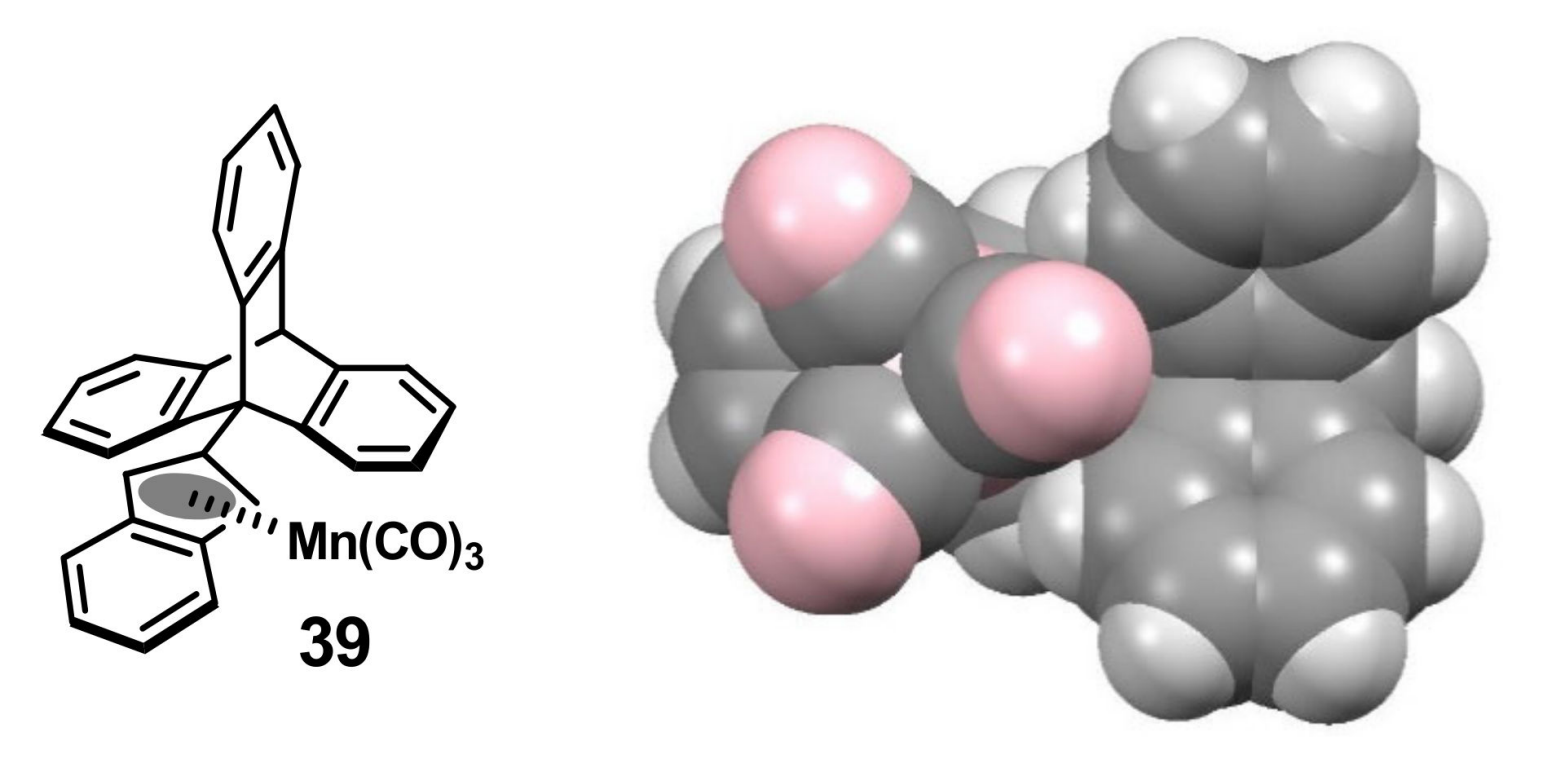
Space-fill view of [η^5^-2-(9-triptycyl)indenyl]tricarbonylmanganese, **39**, showing how a carbonyl ligand is positioned directly between two blades of the triptycene.

The crucial experiment whereby deprotonation of 36 to form its η^5^-haptotropomer, 40, once again brought about a 2:1 splitting of the triptycyl blade NMR resonances is depicted in Scheme 13. The space-filling representation (Figure 17) of the η^5^-Mn(CO)_3_ complex, 39, which is an ideal structural model for the isoelectronic anion, 40, illustrates unequivocally how the bulky organometallic group obtrudes into a valley between two benzo blades, thus hindering paddlewheel rotation. One can only conclude that shuttling of the Cr(CO)_3_ moiety from the six-membered to the five-membered ring of the 2-indenyl component, brought about by deprotonation, represents a pH-dependent organometallic molecular brake [21].

## 3. Ferrocenyl Anthracenes and Triptycenes

### 3.1. Mono- and di-Ferrocenyl Anthracenes

**Scheme 14:**
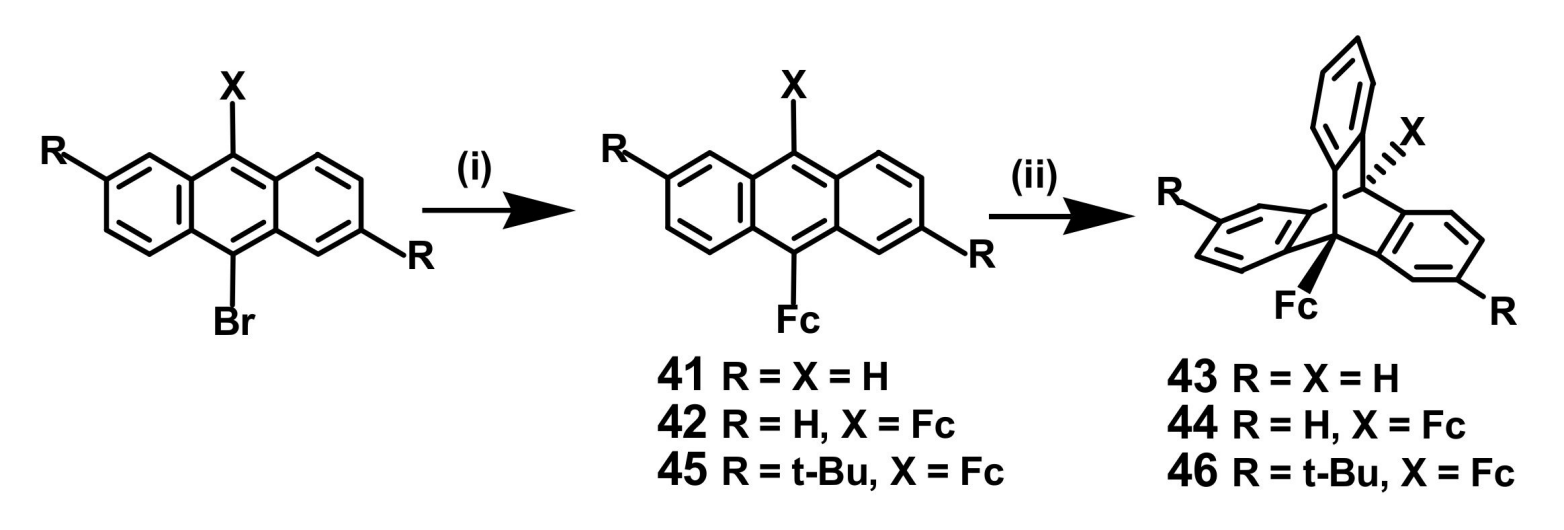
Syntheses of mono- and di-ferrocenyl anthracenes and triptycenes. Reagents and conditions: (**i**) ferrocenylboronic acid, Bu_4_NOH, (dppf)PdCl_2_, 1,4-dioxane, 24 h, 120 °C; (**ii**) *O*-BrC_6_H_4_F, BuLi, toluene, −5 °C.

The demonstration of an organometallic molecular brake involving an η^6^ ⇿ η^5^ haptotropic shift driven by a deprotonation/protonation sequence prompted us to consider the possibility of using an electrochemically-driven redox approach. To this end, we wished to incorporate archetypal bulky redox-active species, viz. one or more ferrocenyl units. The reported synthesis of 9-ferrocenylanthracene, 41, by Butler, utilising the Negishi-type cross-coupling reaction of chlorozincioferrocene with 9-bromoanthracene using (dppf)PdCl_2_ as the catalyst, led to yields of 30–35% [22]. Previous attempts to bring about a Suzuki cross-coupling were unsuccessful unless a large excess of ferrocenylboronic acid was used along with an almost stoichiometric amount of catalyst [23]. However, when tetrabutylammonium hydroxide in 1,4-dioxane was used as the base and (dppf)PdCl_2_ as the catalyst, yields up to 90% were achievable (Scheme 14) [24].

**Figure 18:**
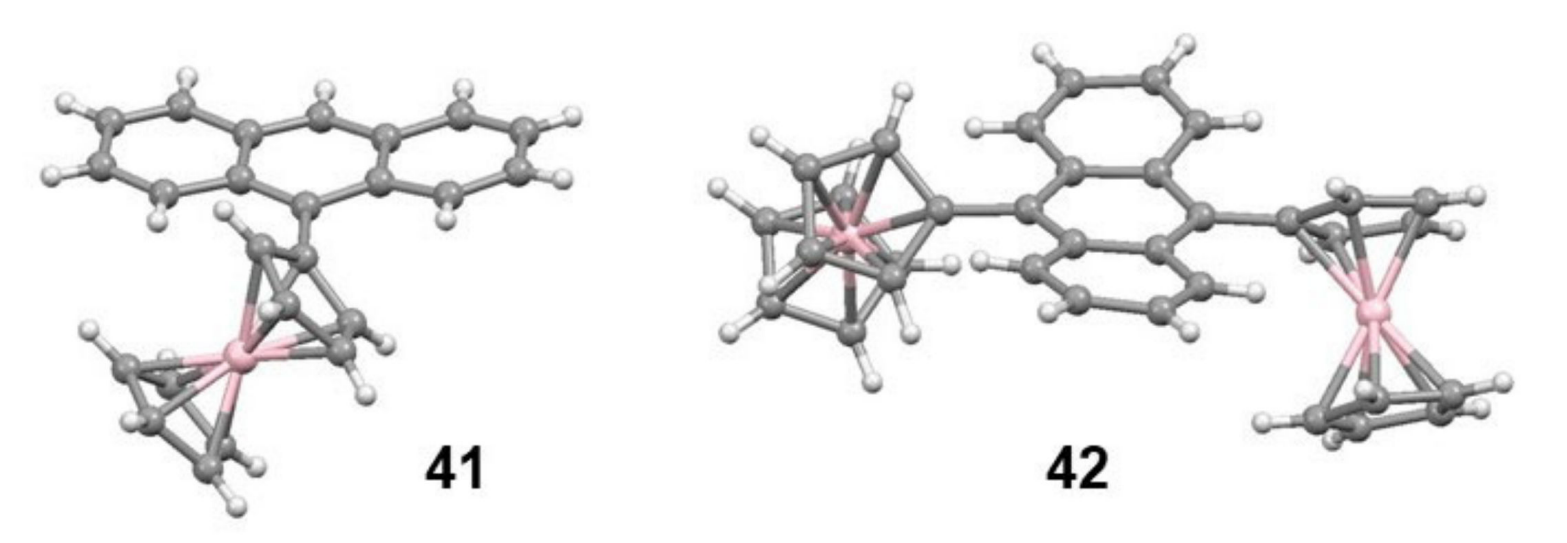
Structures of 9-ferrocenylanthracene, **41**, and 9,10-diferrocenylanthracene, **42**.

As shown in Figure 18, the ferrocenyl substituents in 9-ferrocenylanthracene, 41, and 9,10-diferrocenylanthracene, 42, each make dihedral angles of 45° with the plane of the anthracene; in 42, the ferrocenyls are rotated 89° from each other, thus engendering *C*_2_ symmetry. However, it is evident from our detailed V-T experiments that 42 can exist in two forms, and at 193 K these atropisomers are indeed detectable by NMR in a 38:62 *syn* to *anti* ratio.

The dynamic behaviour of 9-ferrocenylanthracene, 41, paralleled that of [η^5^-2-(9-anthracenyl)indenyl]tricarbonylrhenium, 29, whereby, entirely analogous to that depicted in Figure 12, the system racemises, via a low-energy process, by oscillation of the ferrocenyl group about the mirror plane containing the anthracene, thereby exhibiting dynamic *C*_s_ symmetry. The second, higher-energy, process has a barrier of 44 ± 2 kJ mol^−1^, and together these allow the ferrocenyl moiety to access both faces of the anthracene and both terminal benzo rings; thus, at room temperature, the molecule exhibits effective *C*_2v_ symmetry on the NMR time-scale.

In the structure of 42, depicted in Figure 18, the two ferrocenyl fragments are positioned on the same face of the anthracene, but, because there is a substantial barrier (ca. 45 kJ mol^−1^) towards either of them becoming coplanar with the central anthracene ring, this actually represents an anti-isomer with dynamic *C*_2h_ symmetry. 

### 3.2. Mono- and di-Ferrocenyl Triptycenes

**Figure 19:**
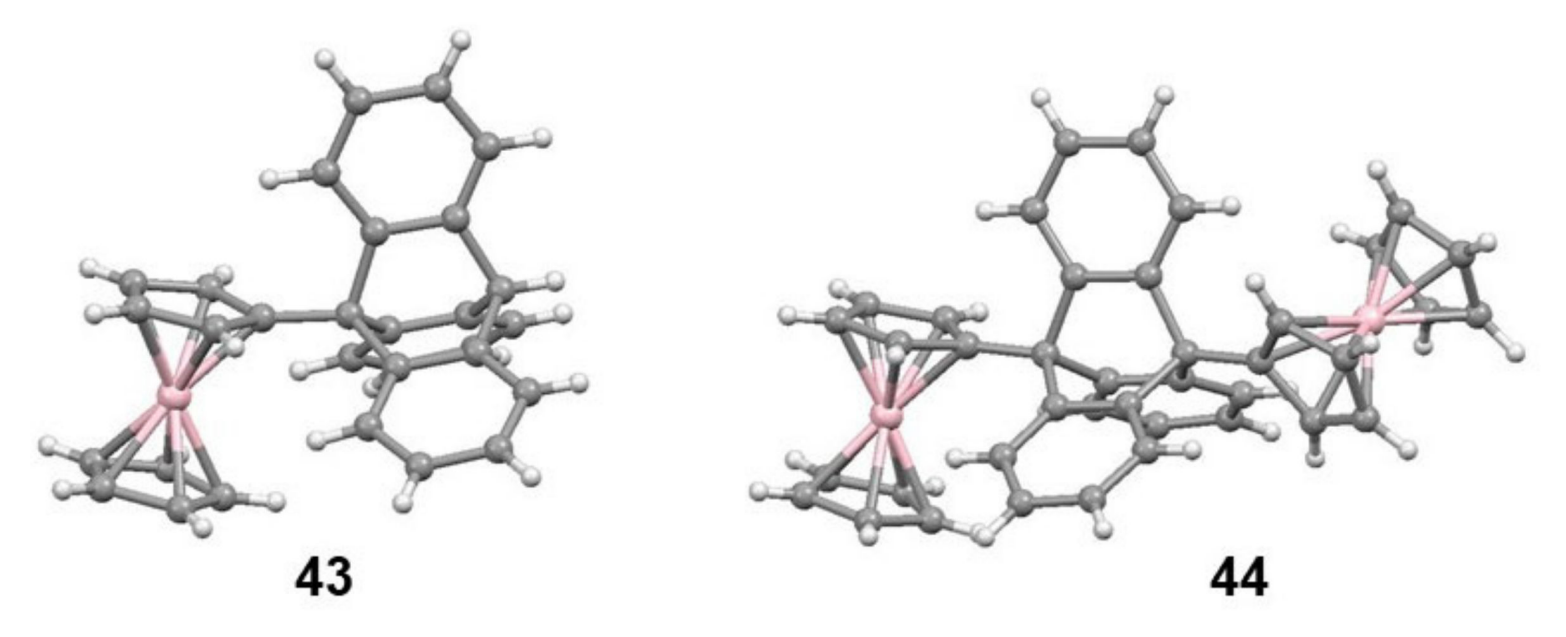
Structures of 9-ferrocenyltriptycene, **43**, and *rac*-9,10-diferrocenyltriptycene, **44**.

Addition of benzyne to 41 and 42 yields the corresponding ferrocenyl triptycenes, 43 and 44, respectively (Figure 19). On a 500 MHz spectrometer, even at room temperature, the signals of the three benzo blades of 43 are split into a 2:1 pattern, indicating slowed paddlewheel rotation on the NMR time-scale; the barrier was evaluated as 69 ± 2 kJ mol^−1^. As with its anthracene precursor, 9,10-diferrocenyltriptycene, 44, exists as two atropisomers, whereby the two sandwich moieties are aligned in the same valley (eclipsed, *C*_2v_, *meso*) or in different valleys (gauche, *C*_2_, *racemic*), and are readily distinguishable by NMR spectroscopy [24].

An Exchange Spectroscopy (EXSY) study of the dynamic behaviour of 9,10-diferrocenyltriptycene revealed a number of interesting features, in particular the nature of the exchange mechanism linking the rotamers of 44. Thus, interconversion of the two mirror forms of the *rac* isomer proceeds in a stepwise manner via the *meso* structure; likewise, a 120° rotation of a single ferrocenyl in the *meso* isomer generates a *rac* structure, and these interconversions are readily detectable in the EXSY experiment when the mixing time is short (50 ms). However, with longer mixing times (300 ms), second generation cross-peaks become evident, thereby revealing exchange between sites within the *rac* or *meso* isomers. This can only occur via the other rotamer, that is, it must follow the sequence *meso* → *rac* → *meso* or *rac* → *meso* → *rac* [24].

**Figure 20:**
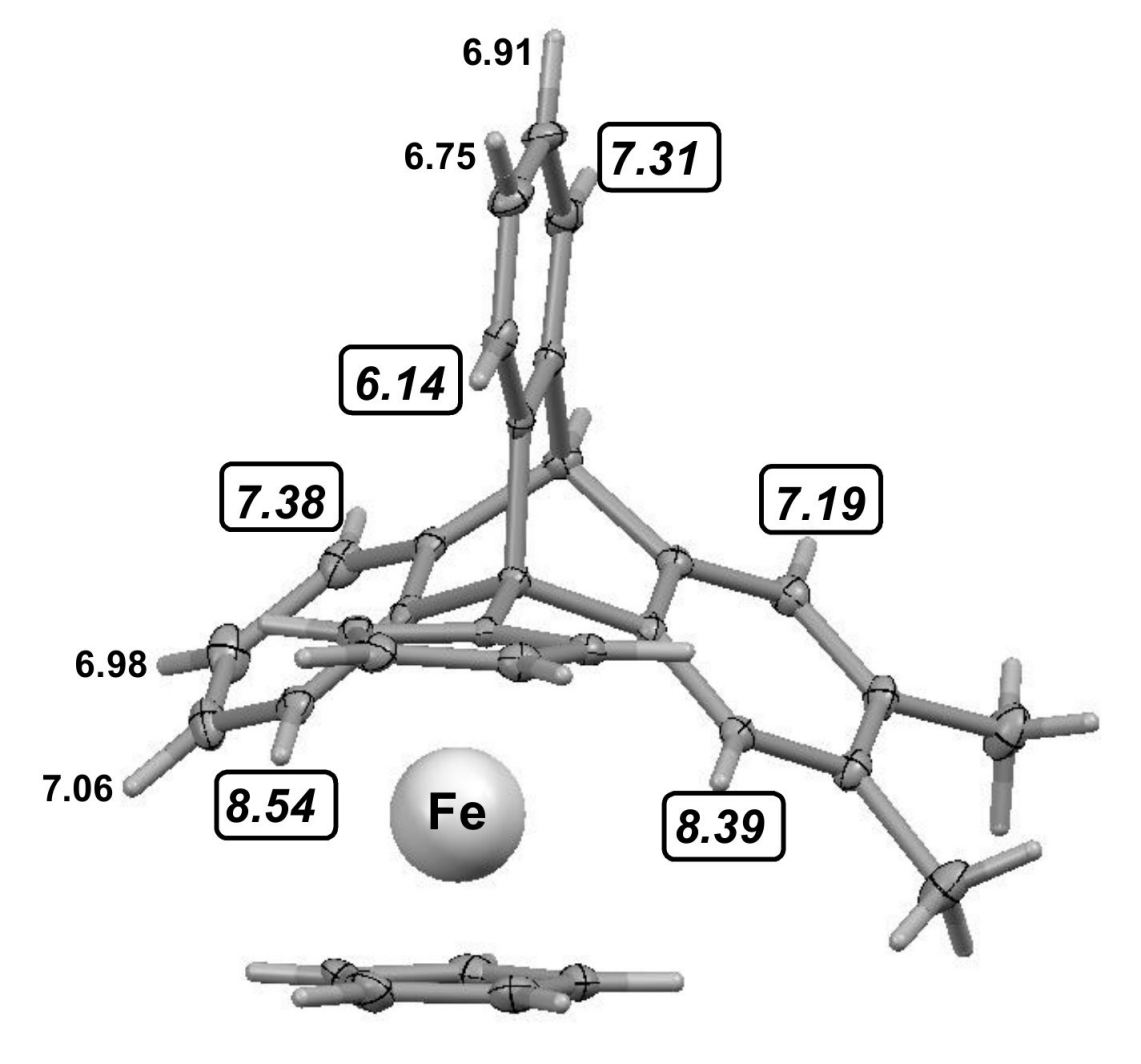
^1^H NMR chemical shifts (ppm) in 2,3-dimethyl-9-ferrocenyltriptycene.

We note that the assignment of ^1^H NMR resonances in the vicinity of a ferrocenyl group is greatly facilitated by its extraordinarily large diamagnetic anisotropy [25]. Typically, in ferrocenyl triptycenes, a proton lying directly above a cyclopentadienyl ring is shielded by ~1.2 ppm relative to its normal aromatic resonance position. In contrast, protons lying near the horizontal plane containing the iron atom are deshielded by ~1.2 ppm, as exemplified by the data for the *C*_1_-symmetric rotamer of 2,3-dimethyl-9-ferrocenyltriptycene whose structure is shown in Figure 20.

**Figure 21:**
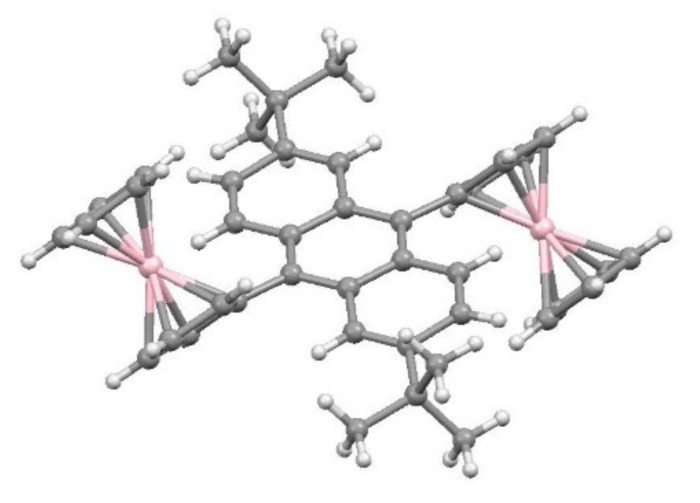
Molecular structure of 2,6-di-tert-butyl-9,10-diferrocenylanthracene, **45**.

This work has been extended to a situation where the triptycene itself is dissymmetric by virtue of the presence of two bulky tert-butyl substituents [26]. Suzuki cross-coupling of 9,10-dibromo-2,6-di-tert-butylanthracene with ferrocenylboronic acid catalysed by (dppf)PdCl_2_ in dioxane gave 45 (Figure 21) in 93% yield, and subsequent Diels-Alder addition of benzyne delivered the required 2,6-di-tert-butyl-9,10-diferrocenyltriptycene, 46 (Scheme 14).

**Figure 22:**
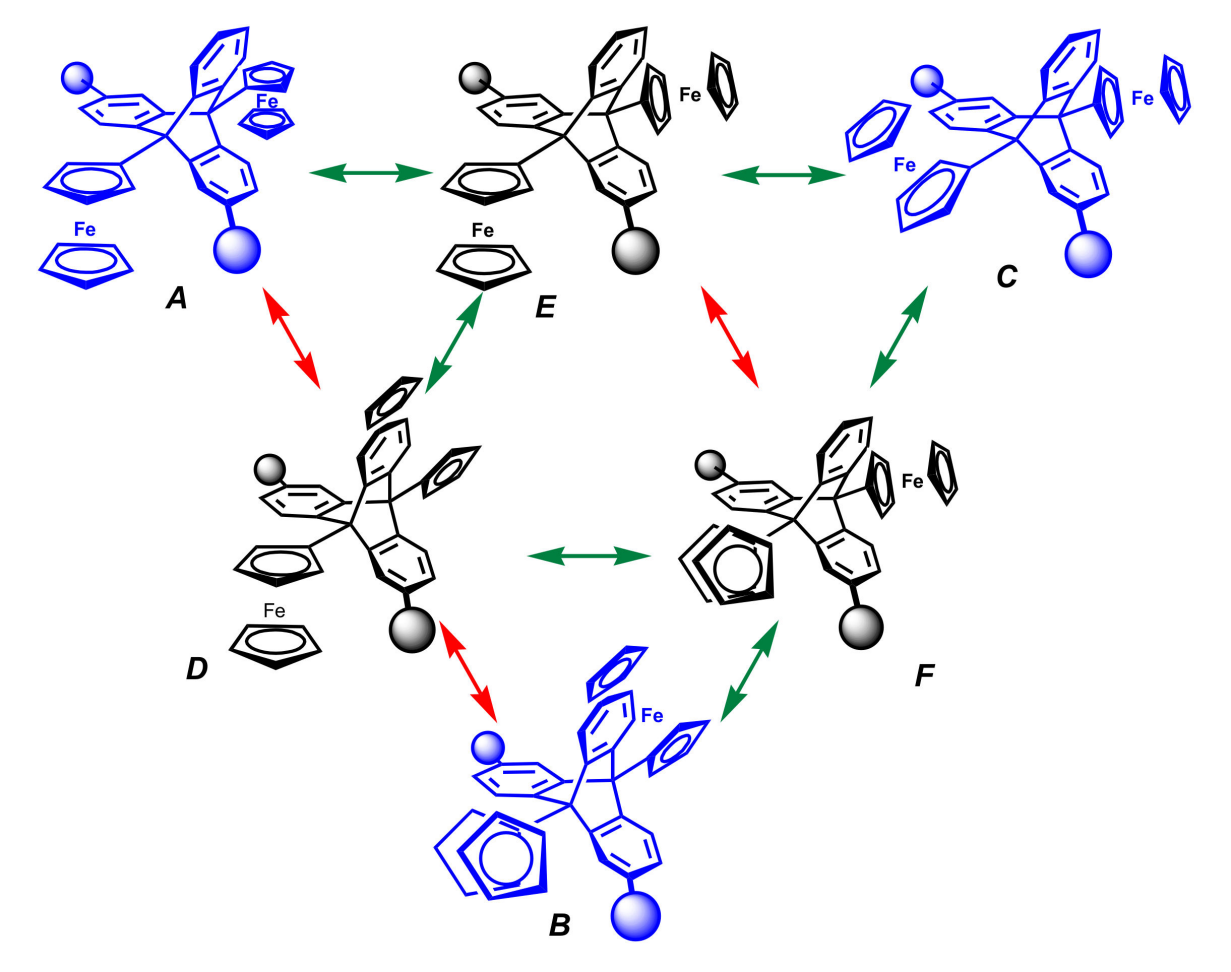
Possible one-step interconversion pathways between conformers of 2,6-di-tert-butyl- 9,10-diferrocenyltriptycene, 46. Green and red arrows indicate high and low barriers, respectively. Structures shown in blue are C_2_-symmetric, and those possessing only C_1_ symmetry are in black [26].

Since each ferrocenyl unit in the triptycene 46 can adopt one of three different positions, one can envisage nine atropisomers: three eclipsed structures and six gauche conformers. However, symmetry considerations lower this number to six different rotamers, three of which are doubly degenerate. Stepwise interconversions of these rotamers do not all have identical barriers, since manoeuvring around a bulky tert-butyl substituent is more sterically demanding than the traversal past a C-H linkage. Nevertheless, as noted above, the diamagnetic anisotropy of the ferrocenyl fragments dramatically enhances the separation between aromatic proton resonances such that, at 600 MHz, the problem becomes resolvable, and all six rotamers can be unequivocally identified. Indeed, in Figure 22, are shown the exchange pathways between the six rotamers; in some cases, direct interconversion between rotamers is possible (such as A ⇿ E), in others a two-step process (e.g., B ⇿ C) is mandatory. Note that the lowest energy route from A to D goes in two steps via E.

### 3.3. Cycloaddition Reactions of Mono- and di-Ferrocenyl Anthracenes

Cycloaddition reactions of anthracenes with a wide range of dienophiles have been intensely studied ever since the original work of Diels and Alder [27], and the relationship between electronic and steric effects is complex. Typically, benzyne adds to 9,10-dimethylanthracene to form exclusively the electronically favoured 9,10-dimethyltriptycene, thus generating three aromatic systems. In contrast, with 9,10-diphenylanthracene, benzyne adds to the 1,4 rather than the 9,10-positions by a factor of 93:7 [28].

**Scheme 15:**
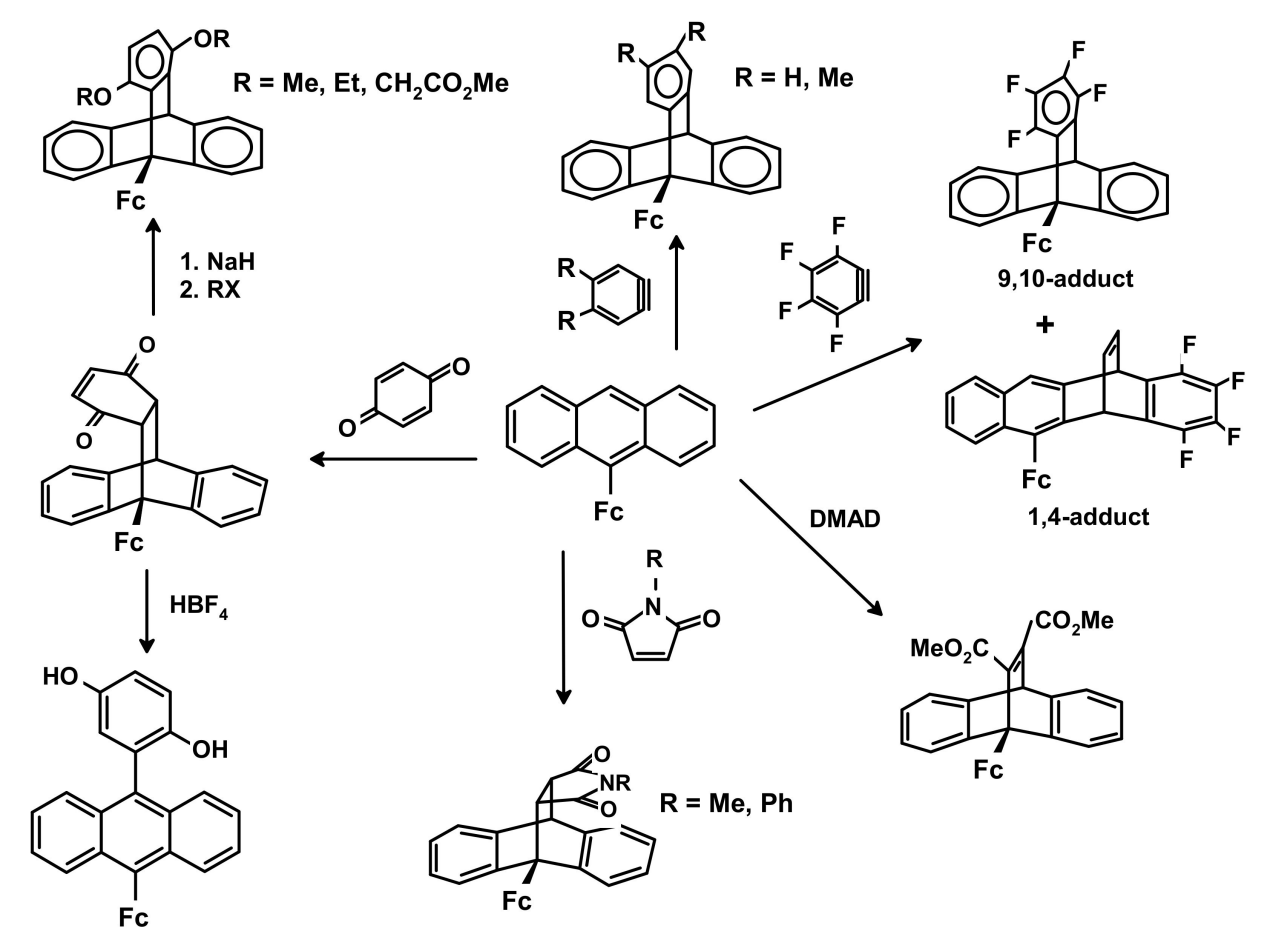
Selected cycloaddition reactions to 9-ferrocenylanthracene.

**Scheme 16:**
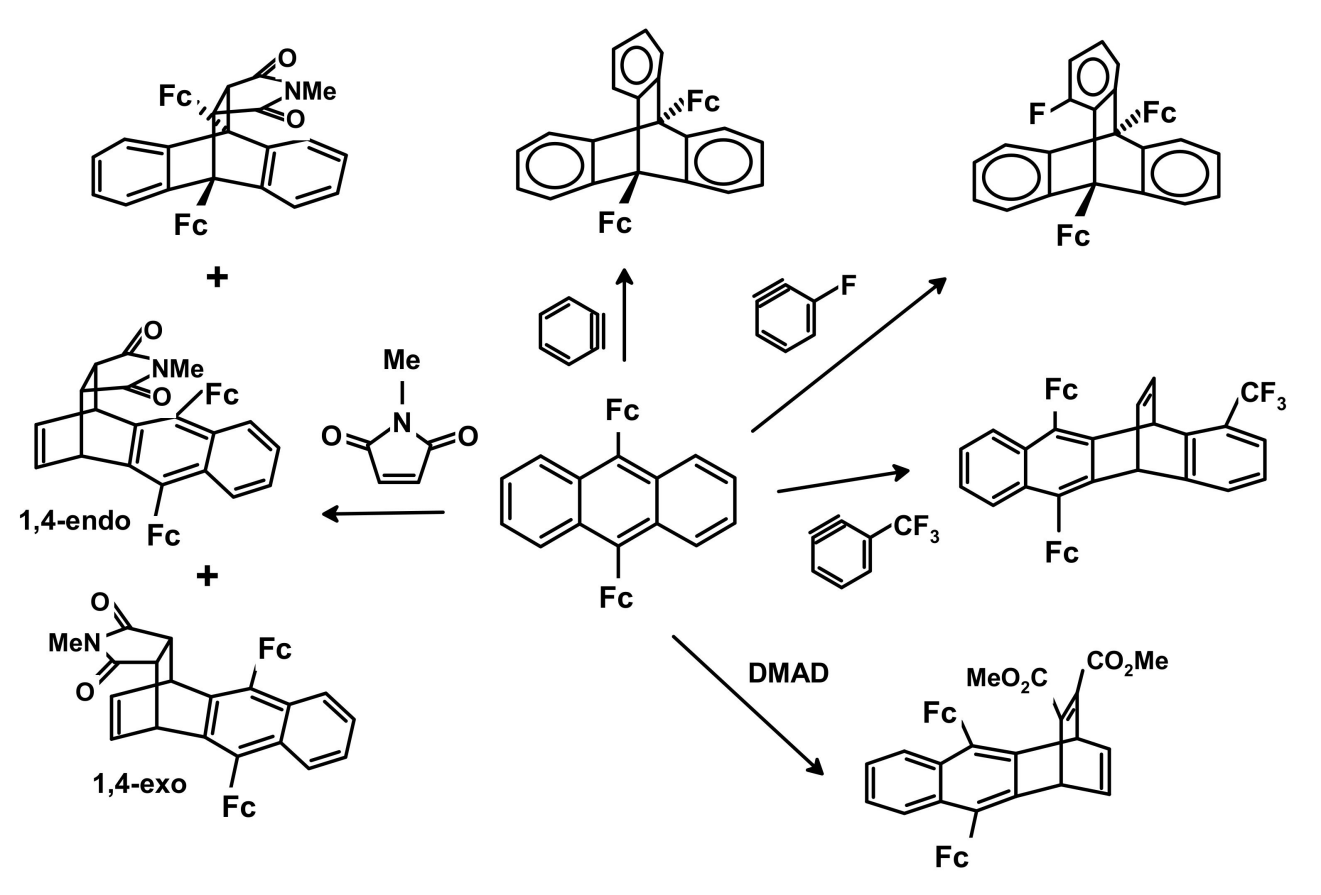
Selected cycloaddition reactions to 9,10-diferrocenylanthracene.

Since palladium cross-coupling procedures had provided us with sufficient quantities of mono- and di-ferrocenyl anthracenes, 41 and 42, to enable further investigation of their reactivity, we undertook a comprehensive study of their cycloaddition chemistry, in particular their reactions with benzynes, alkynes, maleimides and benzoquinone. As shown in Scheme 15, benzyne, dimethylbenzyne and fluorobenzyne add across C(9) and C(10) to form triptycenes, whereas trifluoromethylbenzyne adds in a 1,4-fashion to give the corresponding tetracene, and tetrafluorobenzyne adds both ways. Likewise, DMAD, N-methyl- and N-phenyl-maleimides and benzophenone all react to yield the appropriate barrelene [29,30]. The more sterically hindered 9,10-diferrocenylanthracene also undergoes 9,10-additions with benzynes bearing small substituents, but trifluoromethylbenzyne, DMAD and the maleimides add predominantly at the C(1) and C(4) positions (Scheme 16).

## 4. Syntheses and Dynamic Behaviour of Biindenyls

### 4.1. Cross-Coupling of 2-Phenyl- and 2-Methyl Indenes

**Scheme 17:**
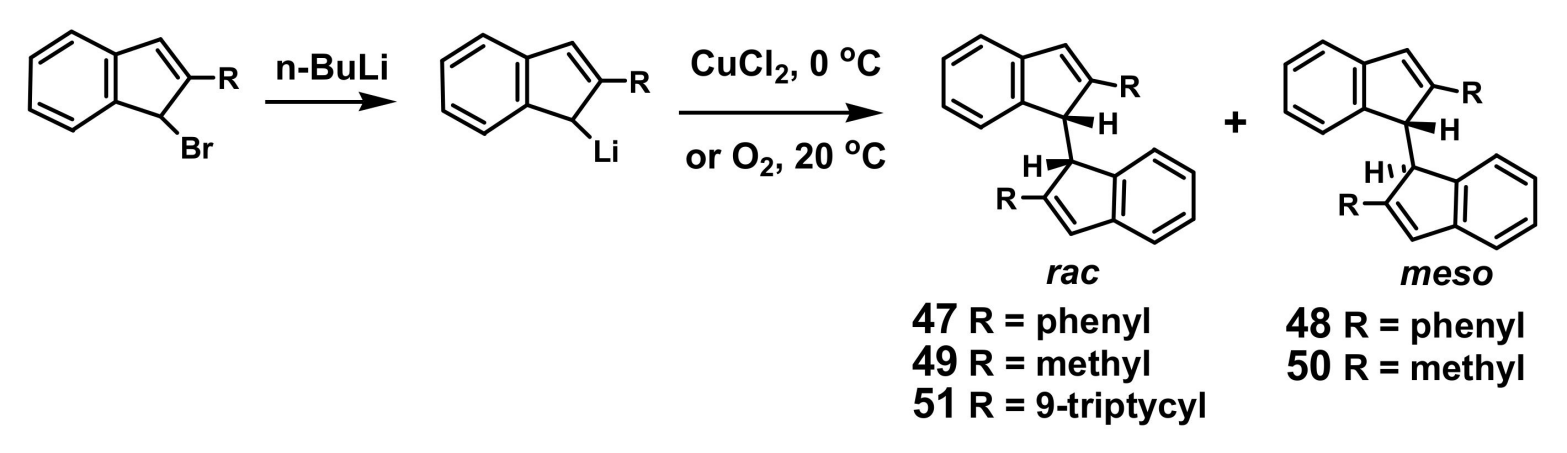
Formation of *racemic* and *meso* bi-indenyls **42**–**45**.

The CuCl_2_-mediated reaction of either 2-phenyl or 2-methylindene generates *racemic* and *meso* biindenyls (Scheme 17) in almost equal amounts, implying a radical process. Surprisingly perhaps, simply allowing their lithio salts to react slowly with oxygen yields only *racemic* products; this has been interpreted in terms of a nucleophilic attack by one anion on the peroxide of its partner, but the mechanism remains speculative [31].

**Figure 23:**
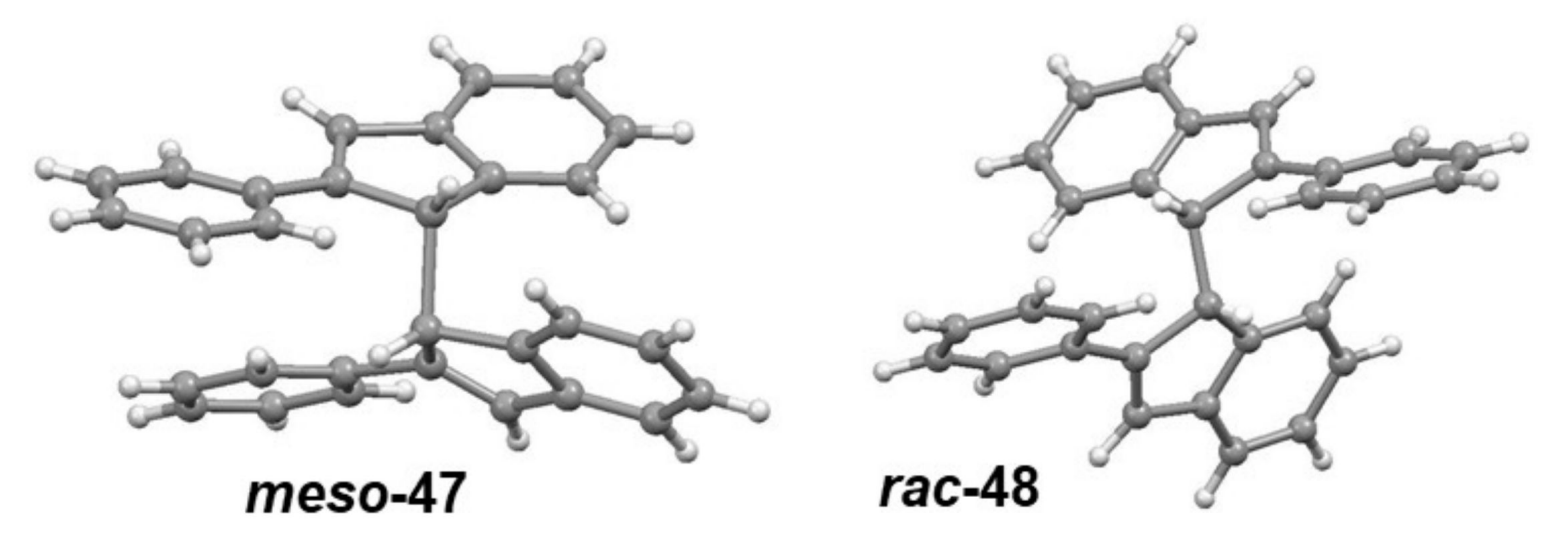
Molecular structures of *meso*- and *rac*-2,2′-diphenyl-1,1′-biindenyl, **47** and **48**.

**Figure 24:**
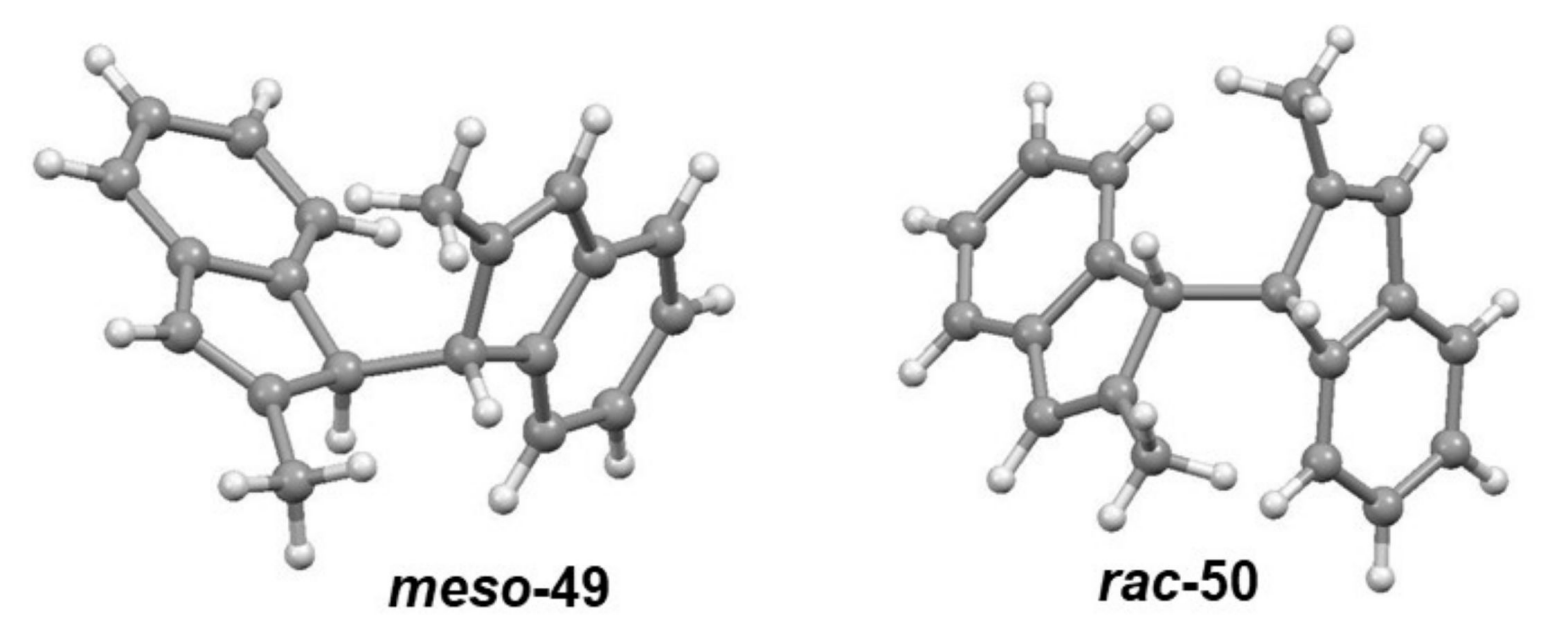
Molecular structures of *meso*- and *rac*-2,2′-dimethyl-1,1′-biindenyl, **49** and **50**.

The structures of molecules 47 through 50 are shown in Figure 23 and Figure 24 and, in all cases, the indenyl fragments adopt a gauche orientation [19,31]. It is interesting to note that the *racemic* products always maintain their *C*_2_ symmetry whatever the rotation angle between the two indenyl units. In contrast, *meso* isomers lose all their symmetry elements unless the central H-C-C-H dihedral angle is 0° or 180°, thereby giving rise either to a mirror plane (*C*_s_) or an inversion centre (*C*_i_), respectively. The rotational barriers about the axis linking the indenyls are ca. 85 and 65 kJ mol^−1^ in the phenyl and methyl cases, respectively [19].

### 4.2. The Curious Case of the 2-Indenyltriptycene Dimer

**Figure 25:**
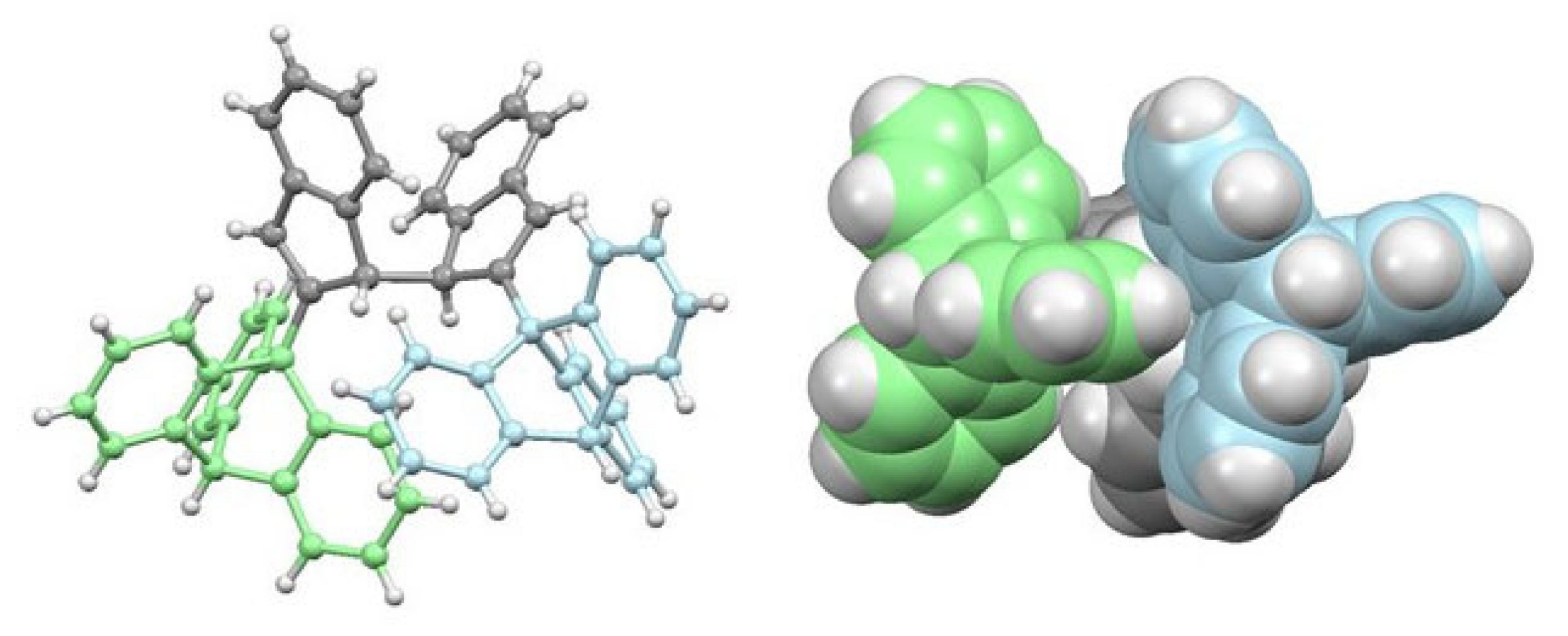
Molecular structure of *rac*-2,2′-di(9-triptycyl)-1,1′-biindenyl, **51**, and a space-fill view showing the gear meshing of the triptycyl groups [31].

In a closely related system, the oxidative coupling of 2-indenyltriptycene produces *rac*-2,2′-di(9-triptycyl)-1,1′-biindenyl, 51, whose structure appears as Figure 25. However, the molecule is not strictly C_2_ symmetric in the solid state; although the 1,1′-biindenyl core maintains its local C_2_ axis, the C_s_-type orientation of the intermeshed triptycene blades breaks this symmetry, as depicted in the space-fill representation. Remarkably, despite the obvious steric congestion, there is no evidence, even at 193 K, of slowed paddlewheel rotation on the NMR time-scale. Evidently, in solution, it behaves as a molecular gearing system that exhibits dynamic C_2_ symmetry implying that the triptycyl units undergo correlated disrotatory motion with concomitant interplanar bending to compensate for the unusually large range of angles (from 103° to 135°) between the paddlewheel blades [31].

## 5. Hindered Rotations in Phenyl-Anthracenes

Many ligands widely used in catalytic asymmetric syntheses, such as BINAP, depend for their activity on the phenomenon of non-interconverting atropisomers that give rise to *R* and *S* enantiomers because of steric hindrance between bulky aromatic ring systems. We were, therefore, intrigued by the computational prediction that 9-phenylanthracene should adopt an orthogonal orientation of the two ring systems and exhibit a rotational barrier of ca. 84 kJ mol^−1^ about the axis connecting them [32]. To probe such an assertion experimentally, one must lower the *C*_2v_ symmetry of 9-phenylanthracene (or the *D*_2h_ symmetry of 9,10-diphenylanthracene), but not introduce any additional steric perturbations. 

**Figure 26:**
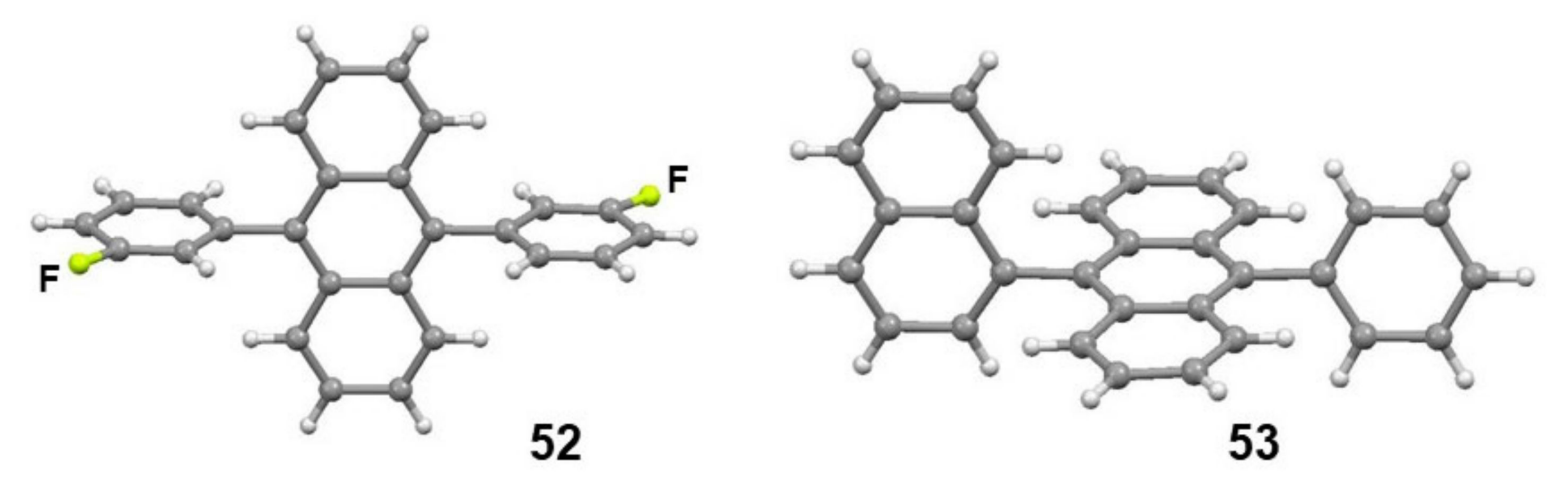
Structures of *anti*-9,10-di(3-fluorophenyl)anthracene, **52**, and of 9-(1-naphthyl)-10-phenylanthracene, **53**.

Once again, a palladium-mediated procedure appeared to be viable. Our initial approach involved the incorporation of *meta*-fluoro substituents in 9,10-diphenylanthracene, via the Suzuki cross-coupling (in 97% yield) of 9,10-dibromoanthracene with 3-fluorophenylboronic acid, catalysed by (dppf)PdCl*_2_* in 1,4-dioxane in the presence of tetrabutylammonium hydroxide [33]. The interconversion of the *syn* and *anti* rotamers of 9,10-di(3-fluorophenyl)anthracene, 52, was monitored by variable-temperature ^13^C-and ^19^F-NMR spectroscopy and yielded a barrier of 88 ± 2 kJ × mol^−1^, gratifyingly close to the calculated prediction. The structure of *anti*-52, appears in Figure 26, showing that in the solid state the fluorophenyl groups are orientated at 85° to the plane of the anthracene.

Nevertheless, even this minor perturbation does not really provide an answer for the unsubstituted phenyl group, which requires that the symmetry of the environment of the phenyl be broken rather than the symmetry of the phenyl itself. This was accomplished by the synthesis and structural characterisation of 9-(1-naphthyl)-10-phenylanthracene, 53, by using the same Suzuki procedure to couple 9-iodo-10-phenylanthracene and 1-naththylboronic acid in 55% yield. Since it is known that the rotational barrier in 9-(1-naphthyl)anthracene is ~160 kJ mol^−1^ [32], this provides a rigid mirror-symmetric framework against which the dynamic behaviour of an unsubstituted phenyl ring can be monitored. The structure of 53 is also shown in Figure 26 and reveals that the dihedral angles of the phenyl and naphthyl rings relative to the plane of the anthracene are 80° and 88°, respectively. Moreover, even at 303 K, on a 600 MHz spectrometer the *ortho* proton resonances of the phenyl ring already show non-equivalence, indicating slowed rotation on the NMR time-scale, and a barrier of at least 85 kJ mol^−1^ [33]. Overall, these observations provide an experimental validation of the computational prediction.

## 6. Conclusions

In our studies of sterically hindered molecules, in particular with their relevance to molecular machines, following the examples of our eminent predecessors, we have taken advantage of the versatile palladium cross-coupling approaches of Stille, Heck, Suzuki and Sonogashira to develop convenient, often high-yield, routes to indenyl and ferrocenyl anthracenes and triptycenes. Their structures, reactivity and dynamic behaviour have been elucidated by X-ray crystallography and variable-temperature NMR spectroscopy and have revealed how rotational barriers between molecular fragments can be manipulated by the controlled migration of organometallic moieties. 

Moreover, copper-catalysed coupling of substituted 2-indenyl systems to form *racemic* and *meso* biindenyls led to an apparently heavily congested molecule, in which, surprisingly, correlated gear rotation of adjacent triptycyl moieties continues unhindered. Finally, the computationally predicted large rotation barrier in 9-phenylanthracene has been experimentally verified by judicious symmetry breaking.

## References

[1] Bruns C.J., Stoddart J.F. (2016). The Nature of the Mechanical Bond.

[2] Dattler D., Fuks G., Heiser J., Moulin E., Perrot A., Yao X., Giuseppone N. (2020). Design of Collective Motions from Synthetic Molecular Switches, Rotors, and Motors. Chem. Rev..

[3] Negishi E. (2011). Magical Power of Transition Metals: Past, Present, and Future (Nobel lecture). Angew. Chem..

[4] Bandera D., Baldridge K.K., Linden A., Dorta R., Siegel J.S. (2011). Stereoselective Coordination of C-5-Symmetric Corannulene Derivatives with an Enantiomerically Pure [Rh-I(nbd*)] Metal Complex. Angew. Chem..

[5] Yokoi H., Hiraoka Y., Hiroto S., Sakamaki D., Seki S., Shinokubo H. (2015). Nitrogen-embedded buckybowl and its assembly with C-60. Nat. Commun..

[6] Yoon I., Benitez D., Zhao Y.-L., Miljanic O.S., Kim S.-Y., Tkatchouk E., Leung K.C.-F., Khan S.I., Goddard W.A., Stoddart J.F. (2009). Functionally Rigid and Degenerate Molecular Shuttles Chem. Eur. J..

[7] Lestini E., Nikitin K., Stolarczyk J., Fitzmaurice D. (2012). Electron Transfer and Switching in Rigid [2]Rotaxanes Adsorbed on TiO_2_ Nanoparticles. Chem. Phys. Chem..

[8] Nikitin K., Stolarczyk J., Lestini E., Müller-Bunz H., Fitzmaurice D. (2008). Quantitative Conformational Study of Redox-Active [2]Rotaxanes, Part 2: Switching in Flexible and Rigid Bistable [2]Rotaxanes. Chem. Eur. J..

[9] Zhu K., Baggi G., Loeb S.J. (2018). Ring-through-ring molecular shuttling in a saturated [3]rotaxane. Nat. Chem..

[10] Harrington L.E., Cahill L.S., McGlinchey M.J. (2004). Toward an Organometallic Molecular Brake with a Metal Foot Pedal: Synthesis, Dynamic Behavior, and X-Ray Crystal Structure of [(9-Indenyl)-triptycene]chromium Tricarbonyl. Organometallics.

[11] Nikitin K., Müller-Bunz H., Ortin Y., McGlinchey M.J. (2007). Joining the rings: The preparation of 2- and 3-indenyl-triptycenes, and curious related processes. Org. Biomol. Chem..

[12] Nifant’ev I.E., Sitnikov A., Andriukhova N.V., Laishevtsev I.P., Luzikov Y.N. (2002). A facile synthesis of 2-arylindenes by Pd-catalyzed direct arylation of indene with aryl iodides. Tetrahedron Lett..

[13] Dang H., Garcia-Garibay M.A. (2001). Palladium-Catalyzed Formation of Aceanthrylenes: A Simple Method for Cyclopentenelation of Aromatic Compounds. J. Am. Chem. Soc..

[14] Wood J.D., Jellison J.L., Finke A.D., Wang L., Plunkett K.N. (2012). Electron Acceptors Based on Functionalizable Cyclopenta[hi]aceanthrylenes and Dicyclopenta[de,mn]tetracenes. J. Am. Chem. Soc..

[15] Lee C.-H., Plunkett K.N. (2013). Orthogonal Functionalization of Cyclopenta[hi]aceanthrylenes. Org. Lett..

[16] Claus T.K., Telitel S., Welle A., Bastmeyer M., Vogt A.P., Delaittre G., Barner-Kowollik C. (2017). Light-driven reversible surface functionalisation with anthracenes: Visible light writing and mild UV erasing. Chem. Commun..

[17] Nikitin K., Müller-Bunz H., Guiry P.J., McGlinchey M.J. (2019). A mechanistic rationale for the outcome of Sonogashira cross-coupling of 9-bromoanthracene and ethynyltrimethylsilane: An unexpected product 4-(9-anthracenyl)-1,3-bis(trimethylsilyl)-but-3-en-1-yne. J. Organometal. Chem..

[18] Rogalski S., Kubicki M., Pietraszuk G. (2018). Palladium catalysed regio and stereoselective synthesis of (E)-4-aryl-1,3-bis(trimethylsilyl)-but-3-en-1-yne. Tetrahedron.

[19] Nikitin K., Fleming C., Müller-Bunz H., Ortin Y., McGlinchey M.J. (2010). Severe Energy Costs of Double Steric Interactions: Towards a Molecular Clamp. Eur. J. Org. Chem..

[20] Nikitin K., Bothe C., Müller-Bunz H., Ortin Y., McGlinchey M.J. (2012). High and Low Rotational Barriers in Metal Tricarbonyl Complexes of 2- and 3-Indenyl Anthracenes and Triptycenes: Rational Design of Molecular Brakes. Organometallics.

[21] Nikitin K., Bothe C., Müller-Bunz H., Ortin Y., McGlinchey M.J. (2009). A Molecular Paddle-wheel with a Sliding Organometallic Latch: Syntheses, X-Ray Crystal Structures and Dynamic Behaviour of [Cr(CO)_3_(η^6^-2-(9-triptycyl)indenene)] and of [M(CO)_3_(η^5^-2-(9- triptycyl)indenyl)] (M = Mn, Re). Chem. Eur. J..

[22] Butler I.R., Hobson L.J., Coles S.J., Hursthouse M.B., Abdul Malik K.M. (1997). Ferrocenyl anthracenes: Synthesis and molecular structure. J. Organometal. Chem..

[23] Vives G., Gonzalez A., Jaud J., Launay J., Rappenne G. (2007). Synthesis of Molecular Motors Incorporating para-Phenylene-Conjugated or Bicyclo[2.2.2]octane-Insulated Electroactive Groups. Chem. Eur. J..

[24] Nikitin K., Müller-Bunz H., Ortin Y., Muldoon J., McGlinchey M.J. (2010). Molecular Dials: Hindered Rotations in Mono and Diferrocenyl Anthracenes and Triptycenes. J. Am. Chem. Soc..

[25] McGlinchey M.J., Nikitin K. (2014). Direct measurement of the diamagnetic anisotropy of the ferrocenyl moiety: The origin of the unusual ^1^H chemical shifts in ferrocenyl-triptycenes and barrelenes. J. Organometal. Chem..

[26] Nikitin K., Muldoon J., Müller-Bunz H., McGlinchey M.J. (2015). A Ferrocenyl Kaleidoscope: Slow Interconversion of Six Diastereomers of 2,6-Di-tert-butyl-9,10-diferrocenyltriptycene. Chem. Eur. J..

[27] Diels O., Alder K. (1931). Synthesen in der hydroaromatischen Reihe VII. Justus Liebigs Ann. Chem..

[28] Klanderman B.H., Criswell T.R. (1969). Reactivity of benzyne towards anthracene systems. J. Org. Chem..

[29] Nikitin K., Müller-Bunz H., McGlinchey M.J. (2013). Diels-Alder Reactions of 9-Ferrocenyl and 9,10-Diferrocenylanthracene: Steric control of 9,10- versus 1,4-Cycloaddition. Organometallics.

[30] Nikitin K., Müller-Bunz H., Ortin Y., McGlinchey M.J. (2011). Different Rearrangement Behaviour of the Cation or Anion Derived from the Diels-Alder Adduct of 9-Ferrocenylanthracene and 1,4-Benzoquinone: Ring-Opening or Paddlewheel Formation. Chem. Eur. J..

[31] Nikitin K., Müller-Bunz H., Ortin Y., Risse W., McGlinchey M.J. (2008). Twin Triptycyl Spinning Tops: A Simple Case of Molecular Gearing with Dynamic C_2_ Symmetry. Eur. J. Org. Chem..

[32] Nori-shargha D., Asadzadeh S., Ghanizadeh F.R., Deyhimic F., Aminic M.M., Jameh-Bozorghi S. (2005). Ab initio study of the structures and dynamic stereochemistry of biaryls. J. Mol. Struct..

[33] Nikitin K., Müller-Bunz H., Ortin Y., Muldoon J., McGlinchey M.J. (2011). Restricted Rotation in 9-Phenylanthracenes: A Prediction Fulfilled. Org. Lett..

